# Chemical Profiles of Two Pheromone Glands Are Differentially Regulated by Distinct Mating Factors in Honey Bee Queens (*Apis mellifera* L.)

**DOI:** 10.1371/journal.pone.0078637

**Published:** 2013-11-13

**Authors:** Elina L. Niño, Osnat Malka, Abraham Hefetz, David R. Tarpy, Christina M. Grozinger

**Affiliations:** 1 Department of Entomology, North Carolina State University, Raleigh, North Carolina, United States of America; 2 W.M. Keck Center for Behavioral Biology, North Carolina State University, Raleigh, North Carolina, United States of America; 3 Department of Zoology, Tel Aviv University, Tel Aviv, Jerusalem, Israel; 4 Department of Genetics, North Carolina State University, Raleigh, North Carolina, United States of America; Université Paris 13, France

## Abstract

Pheromones mediate social interactions among individuals in a wide variety of species, from yeast to mammals. In social insects such as honey bees, pheromone communication systems can be extraordinarily complex and serve to coordinate behaviors among many individuals. One of the primary mediators of social behavior and organization in honey bee colonies is queen pheromone, which is produced by multiple glands. The types and quantities of chemicals produced differ significantly between virgin and mated queens, and recent studies have suggested that, in newly mated queens, insemination volume or quantity can affect pheromone production. Here, we examine the long-term impact of different factors involved during queen insemination on the chemical composition of the mandibular and Dufour's glands, two of the major sources of queen pheromone. Our results demonstrate that carbon dioxide (an anesthetic used in instrumental insemination), physical manipulation of genital tract (presumably mimicking the act of copulation), insemination substance (saline vs. semen), and insemination volume (1 vs. 8 µl) all have long-term effects on mandibular gland chemical profiles. In contrast, Dufour's gland chemical profiles were changed only upon insemination and were not influenced by exposure to carbon dioxide, manipulation, insemination substance or volume. These results suggest that the chemical contents of these two glands are regulated by different neuro-physiological mechanisms. Furthermore, workers responded differently to the different mandibular gland extracts in a choice assay. Although these studies must be validated in naturally mated queens of varying mating quality, our results suggest that while the chemical composition of Dufour's gland is associated with mating status, that of the mandibular glands is associated with both mating status and insemination success. Thus, the queen appears to be signaling both status and reproductive quality to the workers, which may impact worker behavior and physiology as well as social organization and productivity of the colony.

## Introduction

Pheromones are chemicals released by an individual of a species that evoke an innate response in another individual of the same species [1]. They serve as a communication system for many organisms – including yeasts, insects, fish, reptiles, and mammals – and can cause both behavioral (releaser effects) and physiological changes (primer effects) in the receiver (reviewed in [2]). Pheromones are often complex blends of chemicals, and they can serve many functions including aggregation, alarm, food trail marking, and mate attraction. Pheromone production can be modulated by many environmental factors, for example an individual's diet [3], presence of pathogens [4], [5], or pesticide exposure [6]. It can also be affected by an individual's physiological state; mating, for example, profoundly alters pheromone production in females in many sexually reproducing species [3]. These changes in pheromone composition could have significant consequences, and in the case of social insects such as honey bees, alterations in pheromone production could lead to changes in social networks that could potentially have implications for the entire colony.

The honey bee queen is the primary reproductive female in the colony, and she produces pheromones that largely regulate colony social organization [7]. There are multiple pheromone producing glands in the queen [7], but the two best studied are the mandibular glands [8] and Dufour's gland [9], [10]. Though the complete queen pheromone bouquet has not yet been characterized, five active components produced by the mandibular glands have been identified and termed “queen mandibular pheromone” or QMP [8]. These compounds are 9-oxo-2-decenoic acid (9-ODA), both enantiomers of 9-hydroxy-2-(E)-decenoic acid (9-HDA), methyl p-hydroxybenzoate (HOB), and 4-hydroxy-3-methoxyphenylethanol (HVA) [11]. QMP has been found to produce many of the same behavioral and physiological responses in workers as the whole queen pheromone blend. As a releaser pheromone, it induces a retinue response, where workers surround, antennate, and/or lick the queen [11], thereby spreading the pheromone throughout the colony [12]. As a primer pheromone, QMP inhibits worker behavioral maturation [13], increases worker fat stores [14], and alters worker brain gene expression [15]. It also increases foraging activity [16], attracts workers to a swarm [17], and inhibits rearing of new queens [18]. Lastly, QMP inhibits worker ovary activation [19], as well as the associated production of queen-like esters in the Dufour's gland of workers [20].

In comparison, the role of the Dufour's gland in honey bee queens, as well as in most social bees, is not entirely understood [21]. Within Hymenoptera, it is most commonly thought to be involved with production of trail marking pheromones, as well as aggregation, recruitment, sex and queen control pheromones in ants. In solitary bees it appears to have many functions including recognition and nest-marking (reviewed in [21]), while recent evidence suggests a fertility signalling role in a primitively eusocial wasp [22]. The honey bee Dufour's gland contains a mixture of esters synthesized in the gland itself [9] and hydrocarbons that are likely produced by oenocytes and then transported into the gland (reviewed in [21], [23], [24]). It was originally thought that the honey bee queens used Dufour's gland secretions for egg marking in order to avoid removal by policing workers [25], [26]. However, this has since been disputed and new data suggests that it is likely a source of a more general queen signal [27], [28], and it could also signal queen mating quality [29]. Indeed, workers are also attracted to and form a retinue around a queen Dufour's gland extract [30].

After mating, honey bee queens undergo many behavioral, physiological, anatomical, and transcriptional changes [8], [31]–[34], including changes in pheromone profiles. Studies thus far demonstrate that differences in the mating state (e.g., virgin, mated, egg laying; [9], [31], [34], [35]), mating type (e.g., naturally mated vs. instrumentally inseminated; [36], [37]), and mating quantity (e.g., number of drone mates; [29], [33]) have profound effects on queen pheromone production by the mandibular and Dufour's glands. These changes may signal the queen's mating state and quality to workers [8], [31], [34], [35]. However, whether queen pheromone serves as an “honest” signal of queen fecundity (where queen pheromone production is tightly linked to queen reproductive quality and status) or as a form of queen “control” (where the queen produces a pheromone capable of inhibiting worker reproduction regardless of her physiological state) is an ongoing discussion (reviewed in [38]–[41]. It is difficult to experimentally distinguish these two models, and thus far there is evidence to support both in honey bees (discussed in [39], [41]. For example, the studies noted above have found that queen pheromone production is highly variable and modulated by mating state and quality, while other studies [40] have not found significant differences in the blends of pheromones produced by mandibular glands of queens in different reproductive states. Furthermore, many of the studies of mating type or quality have focused on newly mated queens, prior to the initiation of egg-laying. Thus, the long-term effects of variation in the mating process on chemical communication in this system have not been characterized.

Here we manipulate the mating/insemination process to examine the effects of different components of this process on the chemical profiles of both the mandibular and Dufour's glands in queens that have reached their final reproductive state of high ovary activation and egg-laying. In the first experiment, we characterize the effects of carbon dioxide (CO_2_; anesthetic used in instrumental insemination) and physical manipulation of queen genital tract (presumably mimicking copulation) on chemical profiles of the two glands. During natural mating the amount of seminal fluids increases as the number of drones the queen mates with increases (resulting in increased queen mating quality); therefore, in the second experiment we use instrumental insemination as a proxy for natural mating to further dissect the components (insemination substance and volume) contributing to post-mating changes in pheromone production. These studies enable us to determine if specific components of the mating process are critical for regulating pheromone chemical profiles, if the two glands are regulated by common or distinct components (and if they therefore signal similar or different information to workers), and if workers can perceive any resulting differences in chemical profiles and modulate their responses accordingly.

## Materials and Methods

### General Bee Rearing

These studies were performed during the summer of 2007 and 2008 at the Lake Wheeler Honey Bee Research Facility, North Carolina State University (Raleigh, NC). The source colonies used for queen rearing were headed by a queen instrumentally inseminated with semen from a single drone, both of *Apis mellifera carnica* descent (Glenn Apiaries, Fallbrook, CA). Thus the experimental queens were highly related with the average coefficient of relatedness (*G*) of 0.75. Grafting was performed as in [42] and according to [43]. Queens were treated 7 days post-emergence, and at that time they were marked with a marking pen (Dadant and Sons, Inc., Hamilton, IL) and a number tag (Betterbee, Inc., Greenwich, NY) on their thorax. The tip of their left wing was clipped to prevent them from taking mating flights.

We performed two different experiments. In the first experiment in 2007, queens were placed into three treatment groups. In the first group, queens were anesthetized with CO_2_ for 4.0 minutes (CO_2_), the second group was anesthetized with CO_2_ for 4.0 minutes and sham inseminated (the II needle was inserted into the vaginal orifice but the queens were not inseminated with anything; CPM), and the last group consisted of untreated virgins (Virg). These queens were used for the analysis of mandibular gland pheromones. A separate group of queens was treated in the same manner in the summer of 2008, and these queens were used for the analysis of Dufour's gland pheromones.

In the second experiment, queens were placed in one of five groups: untreated virgin control (Virg), instrumentally inseminated with either 1 µl or 8 µl of saline (SA1, SA8), and either 1 µl or 8 µl of semen (SE1, SE8). Saline solution was prepared as in [44]. A mixed pool of semen was prepared from unrelated drones collected at hive entrances upon their return from failed mating flights. Semen was mechanically mixed and used for all inseminations of queens in the SE1 and SE8 groups.

Virgin queens did not receive any treatment, but they were handled, marked, and clipped on the same day as the other queens. After manipulation and/or insemination, queens were returned to their respective mating nuclei (Brushy Mountain Bee Farm, Moravian Falls, NC) which were equipped with queen excluder gates preventing the queens from taking mating flights. The queens remained in mating nuclei for 10 days and they were collected on dry ice in the morning of day 10 after which they were stored in a −80°C freezer until further processing.

Collected queens were dissected and assigned an ovary score of 1–4 as in previous studies [42], [45]. For the mandibular gland analysis, we only used the virgins with ovary scores of 1 and 2 and treated queens with ovary scores of 3 and 4. For the Dufour's gland analysis we used queens with mixed ovary scores regardless of their treatment group. The numbers of queens used for each sample group are detailed in the materials and methods below as well as in the figure legends.

### Gland Preparation

Dufour's glands were dissected on ice and glands of individual queens were extracted in 100 µl of dichloromethane containing 100 ng/sample eicosane as an internal standard [10].

Whole queen heads were lyophilized to facilitate gland dissection, and paired mandibular glands were dissected on dry ice. Glands of individual queens were extracted in 50 µl diethyl ether solvent with 0.4 μg/μl of undec-10-enoic acid as an internal standard [33]–[35], [46]. Glands were extracted for 24 hrs at room temperature and were placed in −20°C freezer until chemical analysis.

### Chemical Analysis of Gland Extracts

Chemical composition of Dufour's gland secretion (numbers of queens used in each sample group are as follows, Exp. 1: Virg  = 5, CO_2_ = 10, CPM  = 11; Exp. 2: Virg  = 10, SA1  = 11, SA8 = 12, SE1  = 9, SE8 = 6) was determined using GC/MS [10] and quantitative analyses were conducted by GC (Varian CP 3800) using a DB-1 fused silica column that was temperature programmed from 150°C (1 min of initial hold) at 5°C/min to 300°C with a final hold of 10 min. Compound quantification was accomplished by peak integration in comparison to the internal standard.

Mandibular gland extracts (numbers of queens used in each sample group are as follows, Exp. 1: Virg  = 5, CO_2_  = 5, CPM  = 6; Exp. 2: Virg  = 7, SA1 = 6, SA8 = 8, SE1 = 5, SE8 = 6) were analyzed by gas chromatography-mass spectrometry (GC/MS) as in previous studies [33], [34]. An aliquot of an extract was silylated with BSTFA overnight, after which 100 µl of hexane was added to each sample [8], [33]. A 1 µl portion of the silylated sample was analyzed with gas chromatography (GC) as well as GC/MS. In the first experiment, we used GC/MS on a Hewlett-Packard (San Fernando, CA) model 6890GC coupled to a model 5973A mass selective detector (MSD) with an electron impact ion source. The GC was equipped with an HP-5MS (5%diphenyl-95% dimethylsiloxane) capillary column (30-m length, 0.25 µm thickness, and 0.25-mm inside diameter, Agilent Technologies, Palo Alto, CA). In the second experiment, we used GC on an Agilent 6890N GC (Agilent Technologies, Santa Clara, CA) with a HP-5MS capillary column according to methods in [33]. Compound identification was achieved by splitless capillary GC/MS using an Agilent 7890 GC and a model 5975C MSD and a HP-5MS capillary column. Mass spectra were compared to the compounds available in the NIST2 library and mass spectra of known mandibular gland compounds. Compound quantification was accomplished by peak integration in comparison to the internal standard.

### Assay of Behavioral Responses to Mandibular Gland Extracts

Workers perform a “retinue response” to live queens or queen pheromones, in which they are attracted over short distances to the queen/pheromone lure and they lick and antennate it [11], [47]. Here, we presented groups of 30 caged worker bees with the mandibular gland extracts of queens from two different treatment groups (performing pairwise comparisons of all the treatment groups for each experiment) to determine their relative attractiveness. For each gland comparison, 8–11 cages were assayed (see below and figure legend for specific numbers of cages used in each comparison). The “retinue assay” has been performed successfully in a number of studies [11], [37], [48]–[54].

One day old bees derived from a colony headed by an SDI queen (Glenn Apiaries, Fallbrook, CA; to control for variance in worker retinue response due to genetic variability [55]) were reared in cages (30 bees/cage) for 5 days as previously described [33], [35]. Since the absence of queen pheromone causes changes in worker physiology [14], [15], [56], [57], we reared workers in the presence of 0.1 queen equivalent (Qeq) of synthetic QMP (Pherotech, Canada) placed on a glass cover slip and allowed to evaporate before it was introduced into a cage. Extracts used for the retinue assay were produced by pooling the extracts of the individual queens in each group, as before [35] and so that 5 µl of extract was equivalent to 0.05 Qeq of QMP. After five days, workers were exposed to 0.05 Qeq of pooled mandibular gland extracts from two different groups of queens on individual slides, as in [35]. In the first experiment, the cages of workers were offered the following choices: virgin and hexane control (n = 8 cages), virgin and CO_2_ (n = 8 cages), virgin and CPM (n = 9 cages), CO_2_ and CPM (n = 9 cages). In the second experiment, the cages of workers were offered the following choices: virgin and hexane control (n = 8 cages), virgin and SA1 (n = 9 cages), SA1 and SA8 (n = 10 cages), SE1 and SE8 (n = 9 cages), SA8 and SE1 (n = 10 cages), SA1 and SE1 (n = 8 cages), and SA8 and SE8 (n = 11 cages).

In each cage, the number of workers antennating and licking each extract-coated slide (defined as the retinue response) was recorded. Observations were repeated every 5 minutes over a 40 minute period for a total of nine observations. The retinue assay was repeated the following day.

### Statistical Analyses

Prior to statistical analyses, the data from the Dufour's and mandibular gland chemical analyses were tested to determine if they meet the assumptions of ANOVA. We tested for normal distribution of the data using the one-sample Kolgomorov-Smirnov test and tested for homogeneity of variances using the Levene's test (IBM SPSS Statistics for Windows, Version 21.0. Released 2012. Armonk, NY: IBM Corp.). Data were accepted as having a normal distribution at the significance level α≥0.05 and accepted as having equal variances at the significance level α≥0.01.

For the Dufour's gland we examined the quantities of esters, hydrocarbons and individual compounds. In Exp 1, these data met the assumptions of ANOVA and were therefore not transformed prior to analysis. In Exp 2, the data on the quantities of esters, hydrocarbons and individual compounds met the ANOVA assumptions only after the log2-transformation. Statistical significance was determined with an ANOVA with treatment as the main factor (JMP 9.0, SAS, Cary, NC).

For the mandibular gland, we examined the proportions of the individual chemical compounds. In Exp 1, the data for HOB, Unk6, Unk 14 and octadecanoic acid and in Exp 2, HOB, Unk 4, 10HDAA, HVA, tetradecanoic acid, decanedioic acid, Unk 13 and octadecanoic acid were either not normally distributed or did not have equal variances. Thus, in order to meet the assumptions of the ANOVA, these data were arcsine square root transformed prior to analysis. Statistical significance was determined with an ANOVA with treatment as the main factor and Tukey's HSD all pairs comparison was used as a post hoc test (JMP 9.0, SAS, Cary, NC). Linear discriminant analysis was performed on the transformed proportions of either all mandibular gland compounds or QMP components only in JMP 9.0.

Data collected for the retinue response were checked for normality and equality of variance as above. In Exp 1 the data were log2-transformed prior to statistical analysis, while in Exp 2 the data met the ANOVA assumptions. Data were averaged across nine observations for each day and statistical analysis was performed for each treatment pair using a mixed-model ANOVA with treatment as the main effect and day as a repeated variable (SAS 9.1.3., SAS Institute Inc., Cary, NC; [35], [55]).

## Results

### Effects on the Dufour's Gland Pheromone

**Figure 1:**
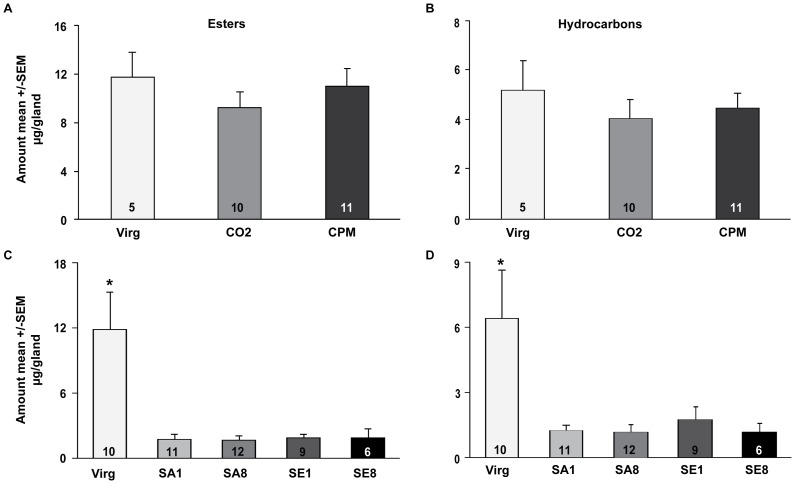
Queen Dufour's gland pheromone production. There was no significant effect of CO_2_ treatment or manipulation on the (**A**) amount of esters produced per queen gland (data not transformed, ANOVA: F_2,23_ = 0.62, P = 0.55) or (**B**) amount of hydrocarbons produced per queen gland (data not transformed, ANOVA: F_2,23_ = 0.43, P = 0.66). There was a significant effect of insemination on (**C**) the amount of esters produced per queen gland (ANOVA on log2-transformed data: F_4,43_ = 9.68, P<0.0001; asterisk indicates significant difference) and (**D**) the amount of hydrocarbons produced per queen gland (ANOVA on log2-transformed data: F_4,43_ = 0.53, P = 0.001). The mean ± SEM is shown for the raw data, numbers in bars denote the number of queens in the group. Abbreviations are as follows: Virg  =  virgin, CO_2_ =  carbon dioxide, CPM  =  CO_2_–treated and physically manipulated, SA1  =  saline 1 µl, SA8 =  saline 8 µl, SE1  =  semen 1 µl, SE8 =  semen 8 µl.

We measured the amount of esters and hydrocarbons contained in the individual queen Dufour's glands for each of the experiments separately. Quantities of esters and hydrocarbons per queen gland as well as ratio of esters to hydrocarbons were log2-transformed when necessary and significant differences among the queens were determined using an ANOVA with treatment as the main dependent variable. In the first experiment, there was no significant effect of CO_2_ (CO_2_ group) and/or physical manipulation (CPM group) on the amount of esters/gland (data not transformed, ANOVA: F_2,23_ = 0.62, P = 0.55; Figure 1A), hydrocarbons/gland (data not transformed, ANOVA: F_2,23_ = 0.43, P = 0.66; Figure 1B), or ratio of esters to hydrocarbons (data not transformed, ANOVA: F_2,23_ = 0.20, P = 0.99; data not shown). In the second experiment, the total amount of esters/gland was significantly higher in virgin (Virg) queens as compared to all inseminated groups (SA  =  saline, SE  =  semen, 1 = 1 µl, 8 = 8 µl inseminated queens; data log2-transformed, ANOVA: F_4, 43_ = 9.68, P<0.0001; Figure 1C). The same was true for the amount of hydrocarbons (data log2-transformed, ANOVA: F_4, 43_ = 5.53, P = 0.001; Figure 1D). No significant differences in ratio of esters to hydrocarbons due to treatment were observed (data not transformed; ANOVA: F_4,43_ = 1.27, P = 0.30; data not shown).

When we compared the data for individual hydrocarbons and esters we did not find any compounds that were only present in one group of queens suggesting instrumental insemination affects overall amounts of esters and hydrocarbons (Tables S1 and S2).

### Effects on the Mandibular Gland Pheromone

We performed a chemical analysis of the mandibular gland extracts of individual queens for each of the experiments separately. Table 1 contains relative proportions of the 27 compounds found in the first experiment. The relative proportions of 7 compounds were significantly different among the three groups (five were lowest in virgins, while 7-hydroxi-octanoic acid and 9-HDA were the highest in virgins). Table 2 contains the relative proportions of mandibular gland compounds for the second experiment. Out of 28 compounds, two unknown (Unk) compounds showed a non-significant trend with highest proportions in the SE8 queens (Unk 11 and Unk 15) while one compound was the lowest in virgins as compared to all the inseminated queens (Unk 10). Retention times and m/z and intensity for the ten most abundant ions for all the unknown compounds can be found in Tables S3 and S4 and fragmentation patterns for all of the compounds used for statistical analyses can be found in Figures S1 and S2.

**Table 1:** Relative proportions of compounds found in mandibular glands of Virg, CO_2_, and CPM queens.

		Virg (n = 5)	CO2 (n = 5)	CPM (n = 6)		
No.	Compound name	mean ± SE	mean ± SE	mean ± SE	F ratio	P values
**1**	Unk 1	0.00232±0.0010	0.00321±0.0010	0.00152±±0.0015	0.73	0.50
**2**	Unk 2	0.00162±0.0005	0.00210±0.0005	0.00188±0.0004	0.28	0.76
**3**	HOB	0.00755±0.0026	0.01108±0.0026	0.00952±0.0024	0.33	0.72
**4**	7OH C8 Acid	0.13248±0.0068^A^	0.11401±0.0068^AB^	0.10174±0.0062^B^	5.70	0.02
**5**	Benzoic acid	0.04506±0.0044	0.04320±0.0044	0.04185±0.0040	0.15	0.86
**6**	Unk 3	0.00234±0.0003	0.00177±0.0003	0.00219±0.0002	1.25	0.32
**7**	Unk 4	0.00369±0.0004	0.00398±0.0004	0.00395±0.0003	0.20	0.82
**8**	9-ODA	0.51498±0.0159	0.49697±0.0159	0.50414±0.0145	0.33	0.73
**9**	HVA	0.00102±0.0002	0.00131±0.0002	0.00143±0.0002	0.90	0.43
**10**	9-oxydecanoic acid	0.00422±0.0004	0.00328±0.0004	0.00443±0.0004	2.69	0.11
**11**	Unk 5	0.00060±0.0001	0.00072±0.0001	0.00068±0.0001	0.57	0.58
**12**	Unk 6	0.00490±0.0002^A^	0.00550±0.0002^AB^	0.00604±0.0002^B^	9.73	0.00
**13**	9-HDA	0.15200±0.0051^A^	0.13742±0.0056^AB^	0.12882±0.0056^B^	4.94	0.03
**14**	10-HDAA	0.01067±0.0010	0.01165±0.0010	0.00994±0.0009	0.84	0.45
**15**	Unk 7	0.00053±0.0001	0.00057±0.0001	0.00064±0.0001	0.39	0.69
**16**	10-HDA	0.11099±0.0134	0.13441±0.0134	0.12212±0.0122	0.77	0.48
**17**	Decanedioic acid	0.00183±0.0003	0.00249±0.0003	0.00178±0.0003	1.53	0.25
**18**	Unk 8	0.00193±0.0003	0.00304±0.0003	0.00297±0.0003	3.92	0.05
**19**	Unk 9	0.00061±0.0001^B^	0.00107±0.0001^A^	0.00071±0.0001^B^	5.39	0.02
**20**	Unk 10	0.00572±0.0011^A^	0.01021±0.0011^B^	0.00802±0.0010^AB^	4.31	0.04
**21**	Unk 11	0.00199±0.0005	0.00366±0.0005	0.00271±0.0004	3.43	0.06
**22**	Unk 12	0.00055±0.0001^A^	0.00088±0.0001^AB^	0.00104±0.0001^B^	4.76	0.03
**23**	Unk 13	0.00044±0.0001	0.00080±0.0001	0.00077±0.0001	3.62	0.06
**24**	Unk 14	0.00103±0.0013	0.00102±0.0013	0.00279±0.0012	0.51	0.61
**25**	Unk 15	0.00389±0.0011^A^	0.00892±0.0011^B^	0.00816±0.0010^B^	6.50	0.01
**26**	cis-13-octadecanoic acid	0.00080±0.0012	0.00423±0.0012	0.00455±0.0011	3.29	0.07
**27**	Octadecanoic acid	0.00084±0.0009	0.00111±0.0009	0.00242±0.0008	0.94	0.42

The mean ± SE is shown for the raw data, but to meet the assumptions of the ANOVA the data for HOB, Unk6, Unk 14, and octadecanoic acid were arcsine square root transformed prior to statistical analysis. Statistical differences in the relative proportions of each individual compound across the three groups of queens were determined using an ANOVA with treatment as the main factor. Post hoc analysis was performed with a Tukey's HSD all pairs comparison and different letters annotate significant differences between groups.

#  =  compound number, Unk  =  unknown, n =  number of queens used for analysis. Retention times and the information on m/z and intensities of the ten most abundant ions for all unknown compounds are available in Table S3 and fragmentation patterns for all of the compounds are available in Figure S1.

**Table 2:** Relative proportions of compounds found in mandibular glands of queens inseminated with different volumes and substances.

		Virg (n = 7)	SA1 (n = 6)	SA8 (n = 8)	SE1 (n = 5)	SE8 (n = 6)		
No.	Compound name	mean ± SE	mean ± SE	mean ± SE	mean ± SE	mean ± SE	F ratio	P values
**1**	Unk 1	0.00158±0.0002	0.00141±0.0002	0.00127±0.0002	0.00098±0.0002	0.00159±0.0002	1.42	0.25
**2**	Unk 2	0.00099±0.0001	0.00098±0.0001	0.00085±0.0001	0.00087±0.0001	0.00090±0.0001	1.49	0.23
**3**	HOB	0.00489±0.0013	0.00613±0.0015	0.00433±0.0013	0.00572±0.0016	0.00574±0.0015	0.23	0.92
**4**	Unk 3	0.00115±0.0001	0.00116±0.0001	0.00098±0.0001	0.00090±0.0001	0.00092±0.0001	1.91	0.14
**5**	7OH C8 Acid	0.07802±0.0043	0.06436±0.0047	0.06778±0.0041	0.05935±0.0051	0.06654±0.0047	2.21	0.09
**6**	Benzoic acid	0.02189±0.0018	0.02337±0.0019	0.02018±0.0016	0.02376±0.0021	0.02512±0.0019	1.15	0.36
**7**	Unk 4	0.00092±0.0002	0.00105±0.0002	0.00098±0.0002	0.00107±0.0002	0.00117±0.0002	0.18	0.95
**8**	Unk 5	0.00348±0.0002	0.00364±0.0003	0.00312±0.0002	0.00311±0.0003	0.00336±0.0003	0.84	0.51
**9**	9-ODA	0.58770±0.0078	0.58393±0.0084	0.57411±0.0073	0.56655±0.0092	0.55666±0.0084	2.36	0.08
**10**	HVA	0.00039±0.0001	0.00057±0.0002	0.00062±0.0001	0.00062±0.0002	0.00071±0.0002	0.68	0.61
**11**	9-oxydecanoic acid	0.00310±0.0003	0.00292±0.0003	0.00338±0.0002	0.00333±0.0003	0.00322±0.0003	0.51	0.73
**12**	Unk 6	0.00639±0.0001	0.00647±0.0001	0.00647±0.0001	0.00641±0.0002	0.00630±0.0001	0.28	0.89
**13**	9-HDA	0.13328±0.0098	0.12519±0.0106	0.14193±0.0092	0.15316±0.0116	0.13878±0.0098	0.90	0.48
**14**	Unk 7	0.00122±0.0002	0.00142±0.0002	0.00119±0.0001	0.00129±0.0002	0.00156±0.0002	1.01	0.42
**15**	10-HDAA	0.00840±0.0015	0.01047±0.0016	0.01092±0.0014	0.01193±0.0017	0.01196±0.0016	1.03	0.41
**16**	Tetradecanoic acid	0.00150±0.0002	0.00148±0.0002	0.00149±0.0001	0.00174±0.0002	0.00195±0.0002	1.47	0.24
**17**	10-HDA	0.08474±0.0079	0.09543±0.0086	0.09211±0.0074	0.09728±0.0094	0.09914±0.0086	0.47	0.75
**18**	Unk 8	0.00086±0.00002	0.00087±0.00002	0.00088±0.00002	0.00083±0.00003	0.00084±0.00002	0.97	0.44
**19**	Decanedioic acid	0.00167±0.0002	0.00185±0.0002	0.00216±0.0002	0.00235±0.0003	0.00231±0.0002	1.80	0.16
**20**	Unk 9	0.00631±0.0004	0.00730±0.0005	0.00682±0.0004	0.00652±0.0005	0.00693±0.0005	0.69	0.60
**21**	Unk 10	0.00725±0.0008^A^	0.00922±0.0008^AB^	0.00941±0.0007^AB^	0.01074±0.0009^B^	0.01029±0.0008^AB^	2.85	0.04
**22**	Unk 11	0.00666±0.0009	0.0080±0.0009	0.00743±0.0008	0.00518±0.0010	0.00891±0.0009	2.12	0.11
**23**	Unk 12	0.00204±0.0001	0.00218±0.0001	0.00216±0.0001	0.00240±0.0002	0.00217±0.0001	0.76	0.56
**24**	Unk 13	0.00175±0.0002	0.00199±0.0003	0.00199±0.0002	0.00138±0.0003	0.00236±0.0003	1.59	0.21
**25**	Unk 14	0.01962±0.0012	0.02209±0.0013	0.02136±0.0011	0.01899±0.0014	0.02323±0.0013	1.75	0.17
**26**	Unk 15	0.01160±0.0008	0.01294±0.0009	0.01264±0.0008	0.01025±0.0010	0.01404±0.0009	2.46	0.07
**27**	Octadecanoic acid	0.00114±0.0004	0.00161±0.0004	0.00162±0.0003	0.00180±0.0004	0.00160±0.0004	0.50	0.74
**28**	Unk 16	0.00146±0.0002	0.00201±0.0002	0.00182±0.0002	0.00151±0.0003	0.00170±0.0002	0.90	0.48

The mean ± SE is shown for the raw data, but to meet the assumptions of the ANOVA the data for HOB, Unk 4, 10HDAA, HVA, tetradecanoic acid, decanedioic acid, Unk 13 and octadecanoic acid were arcsine square root transformed prior to statistical analysis. Statistical differences in the relative proportions of each individual compound across the three groups of queens were determined using an ANOVA with treatment as the main factor. Post hoc analysis was performed with a Tukey's HSD all pairs comparison and different letters annotate significant differences between groups.

#  =  compound number, Unk  =  unknown. Retention times and the information on m/z and intensities of the ten most abundant ions for all unknown compounds are available in Table S4 and fragmentation patterns for all of the compounds are available in Figure S2.

**Figure 2:**
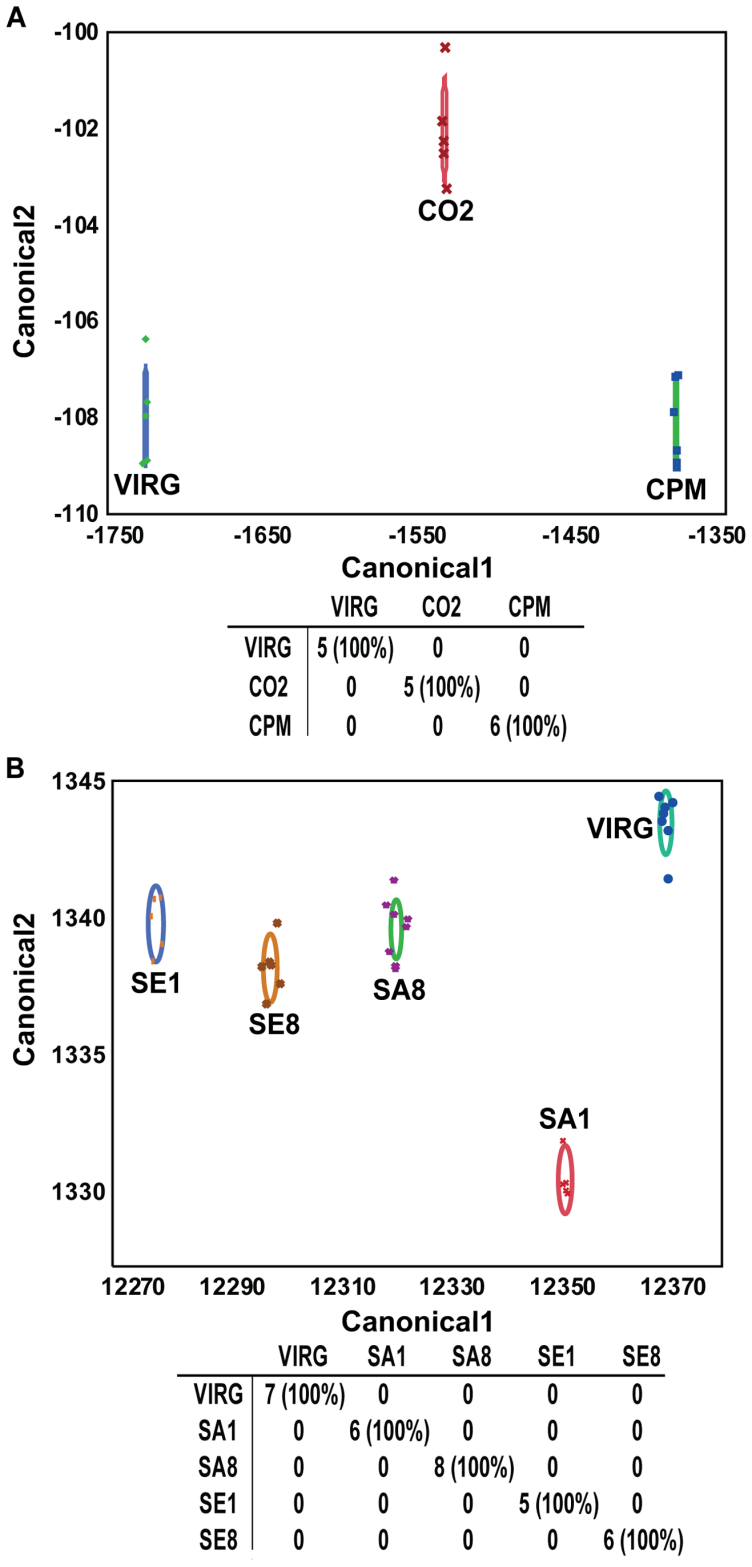
Linear discriminant analysis of all queen mandibular gland pheromone components. The relative proportions of the individual compounds found in the mandibular glands of the queens in experiments 1 and 2 were arcsine square root transformed and subjected to linear discriminant analysis. **A**) Discriminant plot with classification of Virg, CO_2_ and CPM queen groups based on 27 compounds and the table of actual (rows) by predicted (columns) numbers of queens in different groups. Symbols represent individual queens; ellipse represents the 95% confidence region containing the true mean. **B**) Discriminant plot with classification of Virg, SA1, SA8, SE1, and SE8 queen groups based on 28 compounds and the table of actual (rows) by predicted (columns) numbers of queens in the different groups. Numbers in parentheses represent the percentage of queens classified correctly.

**Figure 3:**
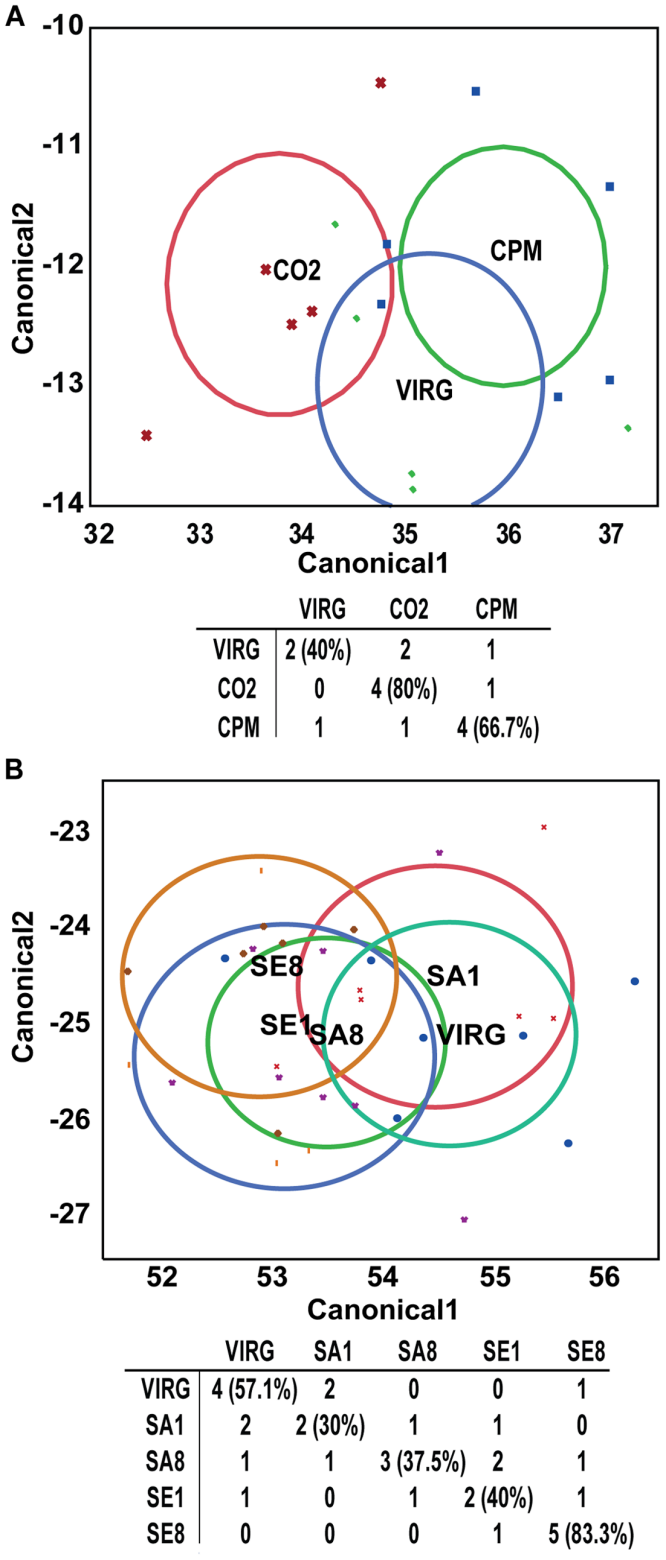
Linear discriminant analysis of QMP components. The relative proportions of the individual compounds found in the mandibular glands of the queens in experiments 1 and 2 were arcsine square root transformed and subjected to linear discriminant analysis. **A**) Discriminant plot with classification of Virg, CO_2_ and CPM queen groups and the table of actual (rows) by predicted (columns) numbers of queens in different groups. Symbols represent individual queens; circle represents the 95% confidence region containing the true mean. **B**) Discriminant plot with classification of Virg, SA1, SA8, SE1, and SE8 queen groups and the table of actual (rows) by predicted (columns) numbers of queens in the different groups. Numbers in parentheses represent the percentage of queens classified correctly.

For both experiments, we performed a linear discriminant analysis on the relative proportions for all mandibular gland compounds (Figure 2). All of the queens were correctly classified according to their treatment group with 100% accuracy, indicating unique chemical profiles for each of the groups. This was not the case when linear discriminant analysis was performed on QMP compounds alone (Figure 3).

### Assay of Behavioral Responses to Mandibular Gland Extract

**Figure 4:**
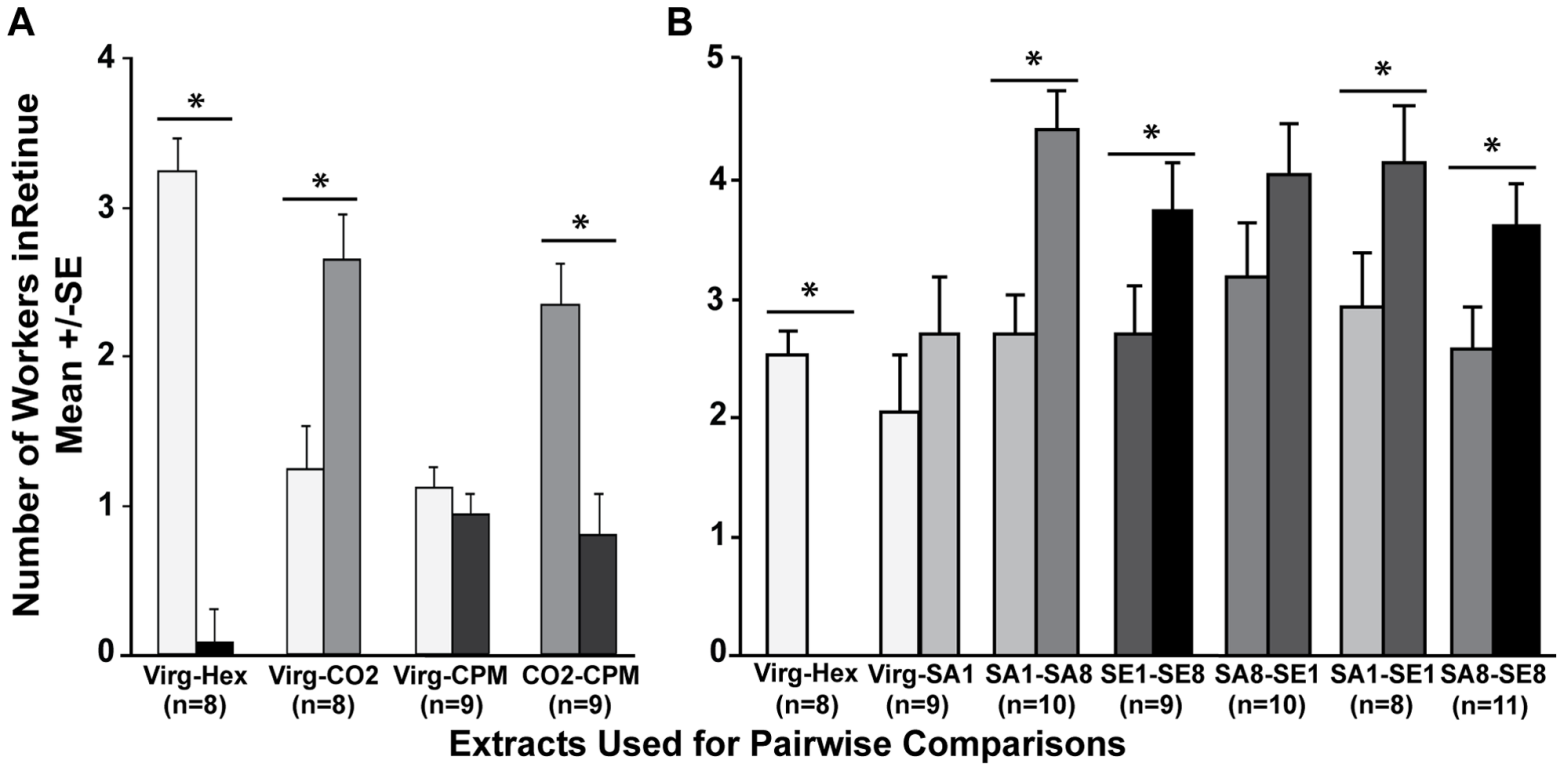
Assay of Behavioral Responses to Mandibular Gland Extracts. Caged groups of 30 five-day-old workers were presented queen mandibular gland extracts from two different queen groups. The number of workers in each cage antennating and/or licking the two extracts were counted every five minutes over a 40-minute period and for two consecutive days. Statistical differences in the preferences for one extract versus the other in the pairwise comparisons were determined via repeated measures ANOVA with treatment as the main effect and day as a repeated variable. The mean ± SE is shown for the raw data, an asterisk above the bars indicates statistically significant differences; n is the number of cages used in comparison. **A**) In the first experiment we tested workers' preference for extracts of Virg, CO_2_, and CPM queens. **B**) In the second experiment we tested workers' preference for extracts of Virg, SA1, SA8, SE1, and SE8 queens.

Workers perform a “retinue response” to live queens or queen pheromone lures, in which they are attracted to the queen/lure over short distances and lick and antennate it [11], [47]. We gave caged worker bees a choice between the extracts of queens from two treatment groups, and monitored the number of bees performing a retinue response to each extract. For each comparison, 8–11 cages of workers with 30 workers per cage were assayed. When the mandibular gland extracts of virgin (Virg), carbon dioxide-treated (CO_2_), and carbon dioxide-treated + physically manipulated (CPM) queens were tested for their attractiveness (Figure 4A), workers preferred CO_2_ to Virg (F_1,7_ = 29.54, P = 0.001) and CPM (F_1,8_ = 41.90, P = 0.0002) extracts. There was no significant difference in worker attractiveness towards Virg vs. CPM queens (F_1,8_ = 0.05, P = 0.84). Workers were significantly more attracted to extracts of Virg than hexane control (F_1,7_ = 248.67, P<0.0001).

When comparing mandibular gland extracts of queens in the second experiment (Figure 4B), workers were most attracted to the extracts of high-volume-inseminated queens (saline 8 µl vs. saline 1 µl: F_1,9_ = 21.80, P = 0.0012; semen 8 µl vs. semen 1 µl: F_1,8_ = 8.00, P = 0.022), and semen-inseminated queens (semen 1 µl vs. saline 1 µl: F_1,7_ = 6.71, P = 0.04; semen 8 µl vs. saline 8 µl: F_1,10_ = 8.85, P = 0.01). Workers also preferred semen 1 µl to saline 8 µl extracts (F_1,9_ = 4.74, P = 0.06), but this difference was not significant at α = 0.05. While workers preferred virgin gland extract over the hexane solvent (F_1,8_ = 95.05, P<0.0001), they were not able to distinguish between extracts from virgin and saline 1 µl queens (F_1,8_ = 4.47, P = 0.07).

## Discussion

In this study, we tested the effects of multiple mating factors on the chemical profiles of two pheromone-releasing glands in honey bee queens. Our results suggest that the chemical composition of these two glands is differentially regulated by factors associated with mating and insemination. While the Dufour's gland pheromone blend was not affected by either CO_2_ or physical manipulation, insemination – regardless of the substance type or volume – reduced the total quantities of esters and hydrocarbons contained in the gland. In contrast, the mandibular gland profiles were affected by both CO_2_ and physical manipulation and further modulated by both insemination substance and volume. Workers were differentially attracted to the mandibular gland extracts of the different groups of queens. Overall, our study demonstrates that (a) individual components of the mating/insemination process can influence pheromone composition even in queens that have fully activated their ovaries, (b) the neuro-physiological mechanisms regulating pheromone biosynthesis seem to respond differently to different components of the mating process, and (c) workers are capable of detecting these differences in the mandibular gland chemical profiles. These results suggest that queens are signaling detailed information about their mating state and reproductive quality to the workers, and the workers are capable of adjusting their behavior accordingly.

The honey bee queen Dufour's gland pheromone consists of both hydrocarbons and wax-type esters [9]. Mating results in a reduced production of esters, while there seem to be no additional effects caused by the onset of egg-laying at least in the honey bee [9]. Our study demonstrates that CO_2_ and physical manipulation do not affect Dufour's gland profiles, but insemination resulted in reduced quantities of esters and hydrocarbons. This suggests that stretch receptors in queen genital tract regulate this process, although the physiological effects do not appear to be dose-dependent. These receptors are likely located in the median or lateral oviducts that expand during insemination, but not in the opening of the genital tract, since physical manipulation had no effect. Though seminal fluid proteins can also play a role in triggering post-mating changes in other female insects [58], [59], they do not appear to regulate Dufour's gland pheromone blend in honey bees since there was no distinct effect of insemination substance. It is worth noting that the expression of *vg*, a gene encoding the egg yolk protein vitellogenin, ten days post treatment, was also not affected by CO_2_ or physical manipulation [42], suggesting a possible co-regulation of these processes.

Unlike the Dufour's gland, chemical profiles of the mandibular glands appear to be sensitive to CO_2_, physical manipulation of the genital tract, insemination volume, and seminal fluids. Thus, the mandibular glands appear to be under the control of both mechanosensory and chemosensory pathways. While the exact mechanism by which these factors regulate changes in pheromone production is not known, it is possible that exposure to CO_2_ triggers changes in pH leading to global physiological changes ([60], discussed in [42]), physical manipulation and insemination volume (via stretch receptors) stimulate mechanosensory neurons in the genital tract causing additional changes, and proteins or other chemical factors transferred with semen cause changes in molecular or physiological signaling pathways. Mechanical stimulation of the genital tract in the females of several moth species causes the cessation in the production of sex pheromone (reviewed in [61]). Similarly, transfer of the accessory gland factors is necessary for inhibition of pheromone production in the female moth species *Helicoverpa zea* [62]. Seminal fluid components might be acting as ligands for receptors located within or outside the female genital tract or might contain compounds with enzymatic activity that cause shifts in female pheromone production after mating [58]. However, the identity of the specific seminal components, as well as their mode of action, is largely unknown not only for honey bees but also for many other economically important insect species, which presents an appealing avenue for future research.

Only a handful of compounds in the mandibular glands were significantly different among the groups of queens. The relative proportion of 9-HDA (one of the components of QMP) was highest in virgins as compared to CO_2_ and CPM. The relative proportion of 9-ODA, the main component of QMP, was also higher (but not significantly) in virgin vs. inseminated queens, with SE8 queens having the lowest proportions. Previous studies in naturally mated queens have demonstrated that quantities of 9-HDA typically increase shortly after mating (∼10 day old), and significantly so in mated, egg-laying queens (five-week-old and two-year-old queens), while 9-ODA quantities remain approximately the same [8]. Al-Qarni et al. [37] demonstrated a decrease in relative proportions of both of these compounds in naturally mated queens two weeks post-mating as compared to queens one week after mating. This suggests that relative levels of 9-ODA and 9-HDA may be sensitive to mating, mating type, and time since mating. Interestingly, relative proportions of 7-hydroxy-octanoic acid were higher in virgins in both experiments, suggesting this compound may also signal a queen's mating state. We also found several currently unidentified compounds that exhibit significant treatment-dependent changes. Future studies will be needed to fully characterize these chemicals and determine if they can modify worker behavior and physiology.

While we did not find many statistically significant differences in individual mandibular gland compounds among different queen groups, a linear discriminant analysis based on relative proportions of all the mandibular gland compounds correctly classified queens into different treatment groups 100% of the time for both experiments, while it was not possible to correctly classify queens based on the QMP components alone. This result is similar to results obtained by Strauss et al. [40], where drone-laying and naturally mated queens could not be separated by linear discriminant analysis based on levels of QMP, 10-hydroxy-2(E)-decenoic acid (10-HDA), and 10-hydroxydecanoic acid (10-HDAA). This suggests that the QMP components do not change rapidly (i.e., within 10 days; also see the above discussion on 9-ODA and 9-HDA) upon insemination, and thus the minor components of the gland may signal mating status and quality in relatively recently mated queens, even after these queens have fully activated their ovaries, as in our study. QMP, thus, may serve primarily as a caste signal that distinguishes queens and workers, or it could signal mating quality in older laying queens.

Workers were also able to discriminate between the mandibular gland extracts of queens based on these overall changes in chemical profiles. Workers preferred extracts of CO_2_-treated queens over both virgin and CPM queens. It is possible that sham-insemination is a more stressful procedure than CO_2_ anesthesia, and this would impair the queens' ability to produce a high-quality pheromone blend. Indeed, in CPM queens there was a trend for several compounds to have relative proportions more similar to those of Virg queens then those of CO_2_ queens (see Table 1). We also previously found that physical manipulation of genital tract causes greater fat body transcriptional changes than CO_2_ alone with several stress-related genes (e. g., *defensin* [63]) being up-regulated in this group [42]. In the second experiment, workers preferred extracts of semen- vs. saline-inseminated queens, and high-volume vs. low-volume inseminated (i.e. well-mated/high mating quality) queens. This suggests that insemination counteracts any negative effects of physical manipulation on the production of an attractive queen pheromone blend. Overall, it is clear that workers are able to detect changes in complete pheromone profiles, even though changes in levels of individual compounds are not significantly different.

The ability of a worker to distinguish between queens of different mating states and reproductive potential may be adaptive. Queen mates only once in her lifetime with an average of 12 drones [64], and several studies have demonstrated that higher genetic diversity within a colony reduces a chance of infectious diseases and improves overall colony productivity which is beneficial for colony homeostasis [65]–[69]. Thus, it could be advantageous for workers to distinguish between poorly mated (inseminated by only a few drones) and well-mated queens (inseminated by many drones). Since mating number is directly associated with insemination volume, this could serve as a suitable proxy for measuring mating number. Our studies indicate that queens of different mating quality produce different pheromone blends, and that workers can distinguish between these blends in a caged choice test and are more attracted to blends of queens inseminated with higher volumes of semen (as also observed in [33]). Furthermore, workers in colonies headed by queens inseminated with high volumes of semen produced significantly fewer esters in their Dufour's glands (an indicator of ovary activation) than workers maintained in colonies headed by low volume inseminated queens, suggesting that queen insemination volume may be associated with differences in altruistic behavior and social organization in the colony [70].

The plasticity of queen pheromone production and its association with mating state and quality suggests that it serves as an honest signal of queen reproductive potential rather than a control mechanism [38], [39], [41]. Our results suggest that honey bee queens signal their mating status through Dufour's gland pheromones, while both their mating status and quality is advertised through the minor components of the mandibular gland pheromone [33], [35], [40], though QMP levels may change over longer timescales [8]. Similar evidence for a fecundity signal has been found in ant queens [71], [72]. Previous work examining effects of queen insemination volume on worker behaviour and physiology in field colonies where queen has been egg-laying for at least two months indeed shows significant differences in pheromone-mediated queen-worker interactions between queens inseminated with low vs. high volumes of semen [70]. However, since there is an effect of the insemination procedure itself and also honey bee queens live on average 2–3 years, an important natural test of this model would be to evaluate the differences in queen pheromone production and worker responses in naturally mated queens and over a longer period of time. Furthermore, our behavioral assays consisted of simply monitoring worker attraction to the mandibular gland; a more proper test would be to examine worker ovary activation rates in the presence of these queens. Indeed, worker ovary activation is higher in colonies headed by unmated virgin and drone-layer queens than mated or inseminated laying queens [73]. However, we and others have noted that it can be challenging to distinguishing between a control and honest signaling system in a highly derived species such as the honey bee [38], [39], and thus additional studies in more primitively social species are necessary to fully understand the mechanisms mediating the evolution of chemical communication.

Overall, our results demonstrate that pheromone composition of queen Dufour's and mandibular glands is under the influence of multiple mating/insemination factors, and that it is likely regulated by different neuro-physiological mechanisms (mechanosensory, chemosensory). While changes in the Dufour's gland pheromones (regulated by insemination volume and substance, but not CO_2_ and physical manipulation) seem to signal queen mating status, the mandibular gland pheromone profiles (regulated by CO_2_, physical manipulation, insemination volume, and insemination substance) are likely a signal of both queen mating status and quality. More importantly, workers are sensitive to differences in mandibular gland pheromones and are more attracted to blends of “well-mated” queens. This suggests that pheromones in queens are acting as an honest signal of queen insemination success and potentially mating quality, even though not all of the components are altered (and particularly those that comprise QMP). There are likely many other factors that could alter pheromone production, such as drone quality (e.g., [74], queen reproductive potential (e.g., [75]), and exposure to pesticides and diseases (e.g., [76]; but see [4]), and these factors remain to be investigated.

## Supporting Information

Figure S1 **Fragmentation patterns of individual compounds in experiment 1.** Shown here are the compounds recorded from the mandibular gland extracts of the queens exposed to CO_2_ and CO_2_+ physical manipulation (fragmentation patterns are shown for one of the CO_2_ queens).
(PDF)

Figure S2 **Fragmentation patterns of individual compounds in experiment 2.** Shown here are the compounds recorded from the mandibular gland extracts of the queens instrumentally inseminated with low or high volume of either saline or semen (fragmentation patterns are shown for one of the SA1 queens).
(PDF)

Table S1 **Quantities of individual hydrocarbons and esters found in the Dufour's glands of the queens exposed to CO_2_ and CO_2_+ physical manipulation.**
(XLSX)

Table S2 **Quantities of individual hydrocarbons and esters found in the Dufour's glands of the queens instrumentally inseminated with low or high volume of either saline or semen.**
(XLSX)

Table S3 **Information for the ten most abundant ions (m/z and intensities) for each of the unknown compounds found in the mandibular gland extracts of the queens exposed to CO_2_ and CO_2_+ physical manipulation.**
(XLSX)

Table S4 **Information for the ten most abundant ions (m/z and intensities) for each of the unknown compounds found in the mandibular gland extracts of the queens instrumentally inseminated with low or high volume of either saline or semen.**
(XLSX)
