# Supplementary material for: Chemical Profiles of Two Pheromone Glands Are Differentially Regulated by Distinct Mating Factors in Honey Bee Queens (Apis mellifera L.)
Source: PLoS One. 2013 Nov 13;8(11):e78637. doi: 10.1371/journal.pone.0078637 (PMC3827242; doi:10.1371/journal.pone.0078637)

Figure S1. Fragmentation patterns of individual compounds in experiment 1. Shown here are the compounds recorded from the mandibular gland extracts of the queens exposed to CO_2 and CO_2+physical manipulation (fragmentation patterns are shown for one of the CO_2 queens).

Compound 1 (8.50min): Unk1

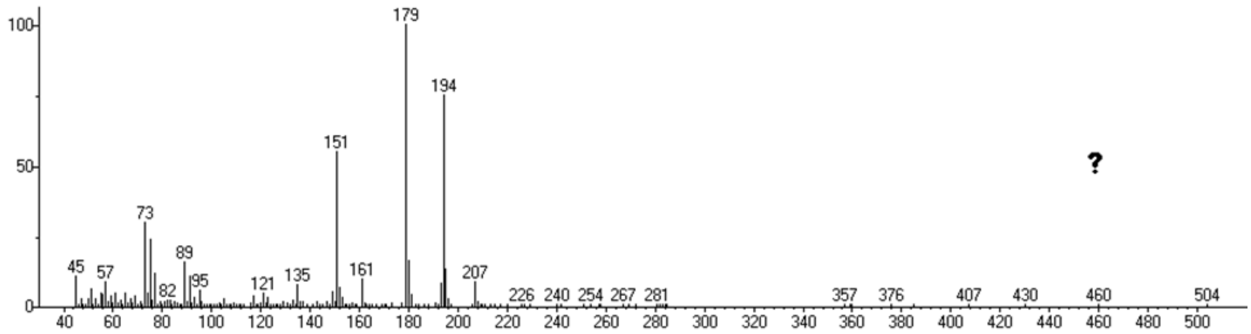

Compound 2 (9.21min): Unk2

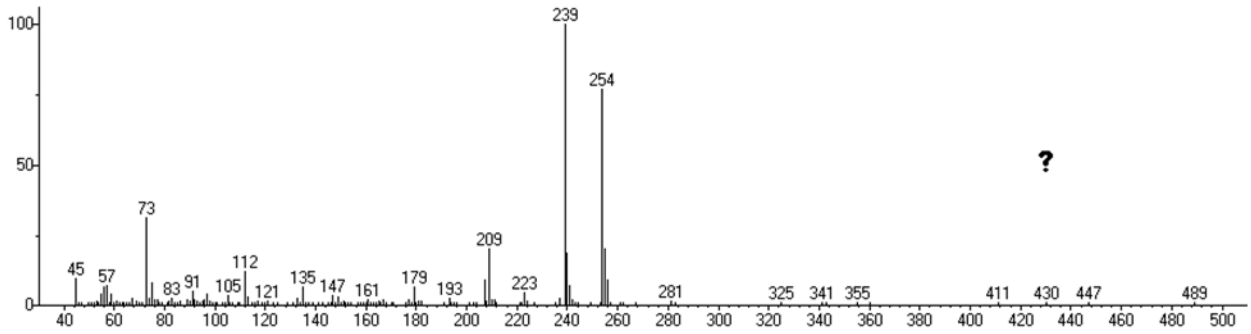

Compound 3 (11.17min): HOB

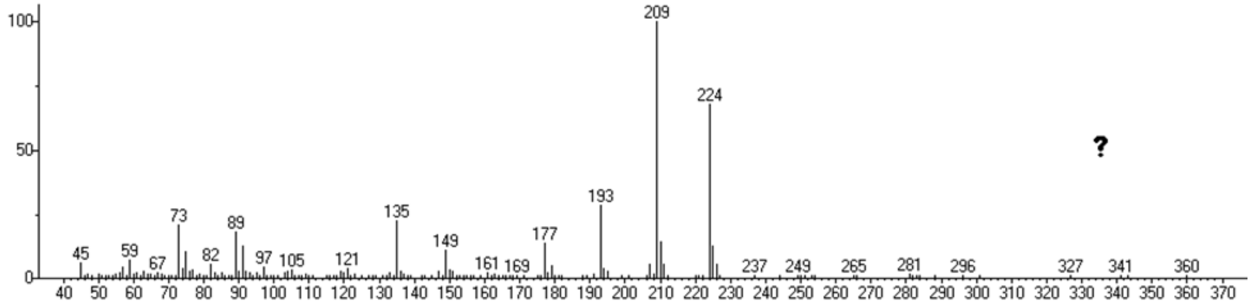

Compound 4 (14.02min):7OH-octanoic acid

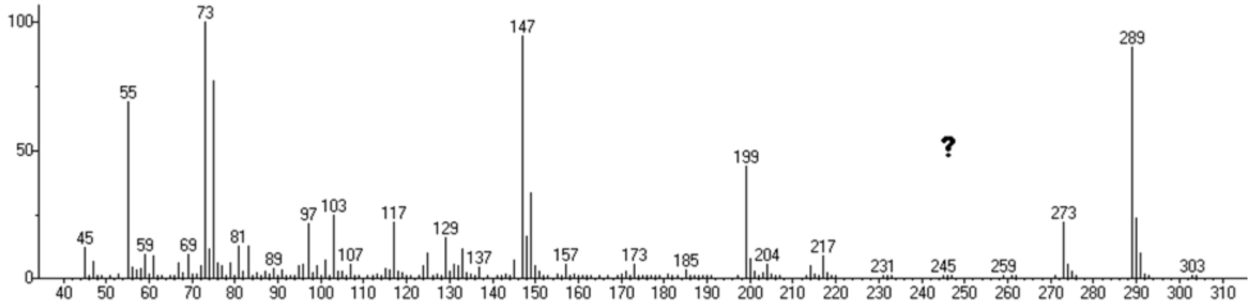

Compound 5 (14.18min): Benzoic acid

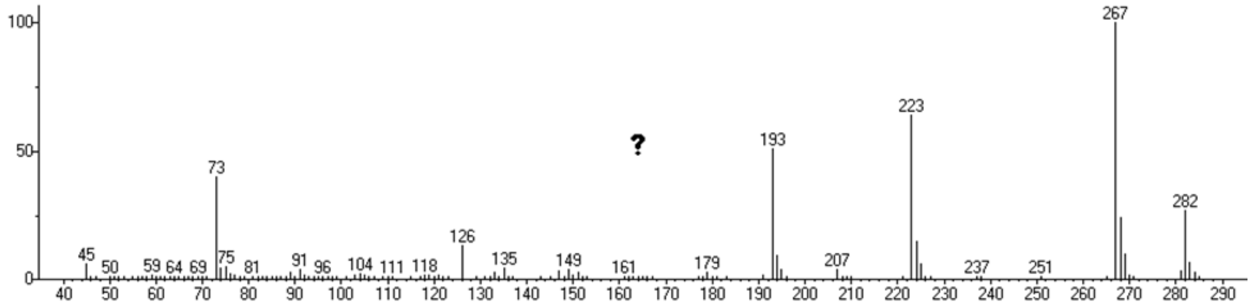

Compound 6 (14.60min): Unk3

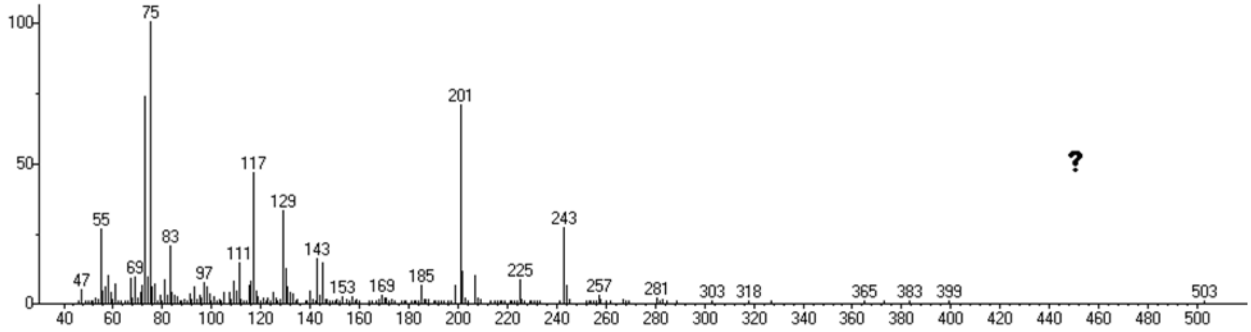

Compound 7 (15.07min): Unk4

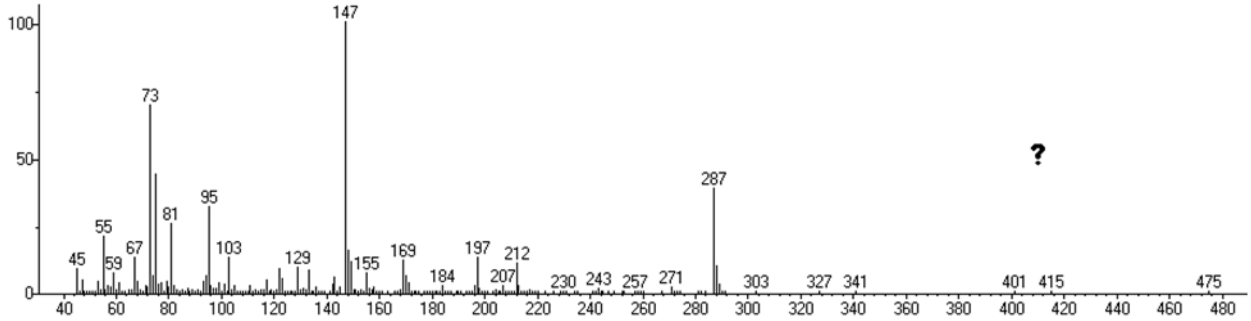

Compound 8 (15.92min): 9-ODA

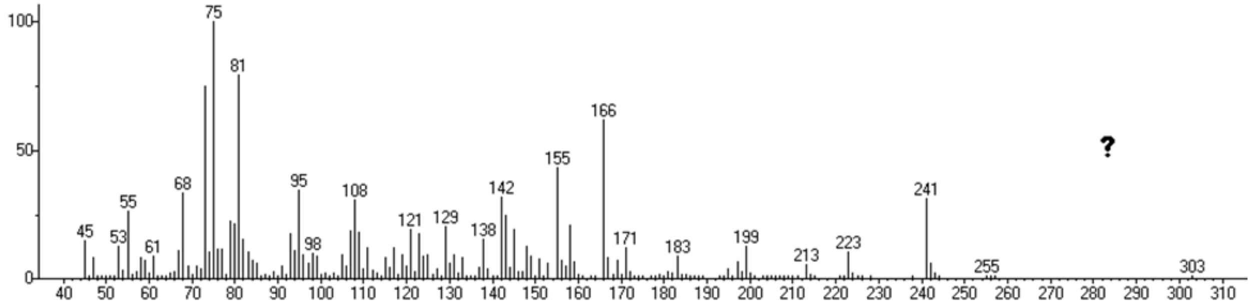

Compound 9 (16.04min): HVA

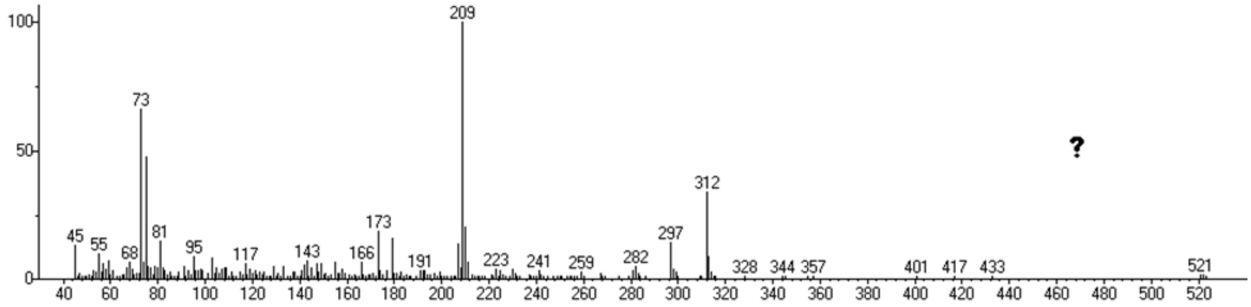

Compound 10 (16.61min): 9-oxydecanoic acid

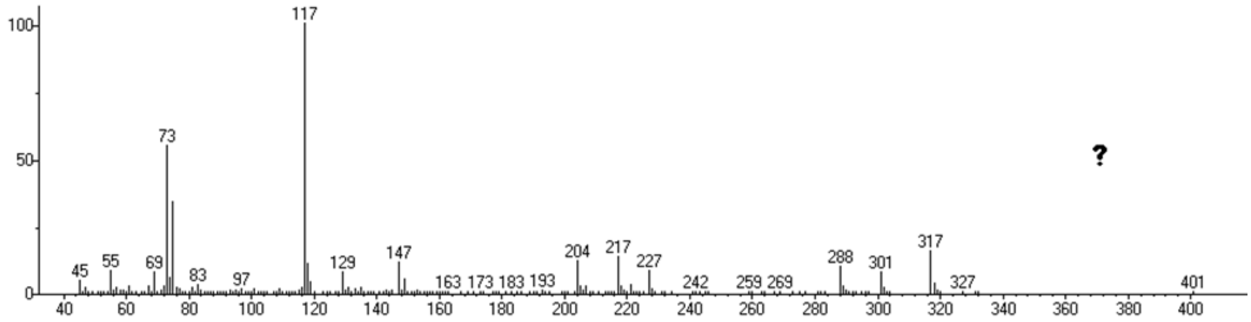

Compound 11 (16.82min): Unk5

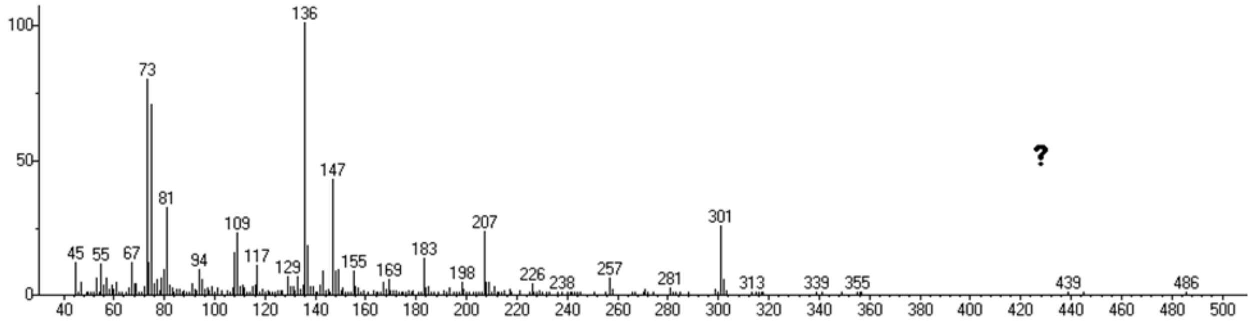

Compound 12 (17.35min): Unk6

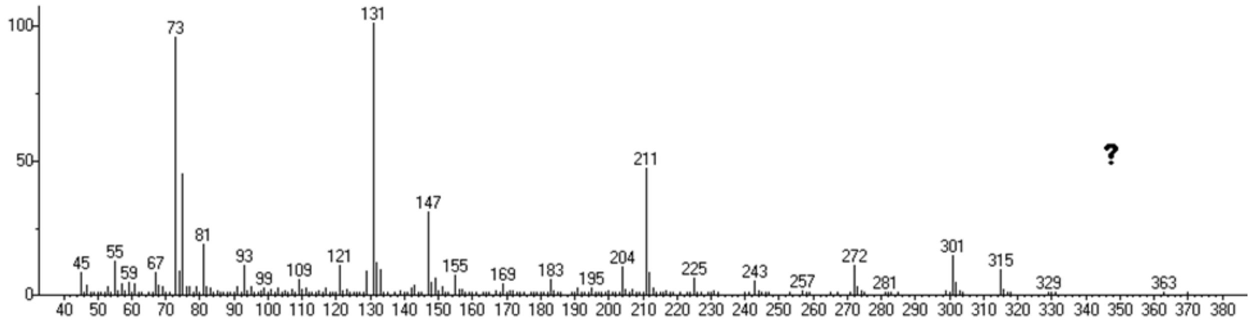

Compound 13 (17.69min): 9-HDA

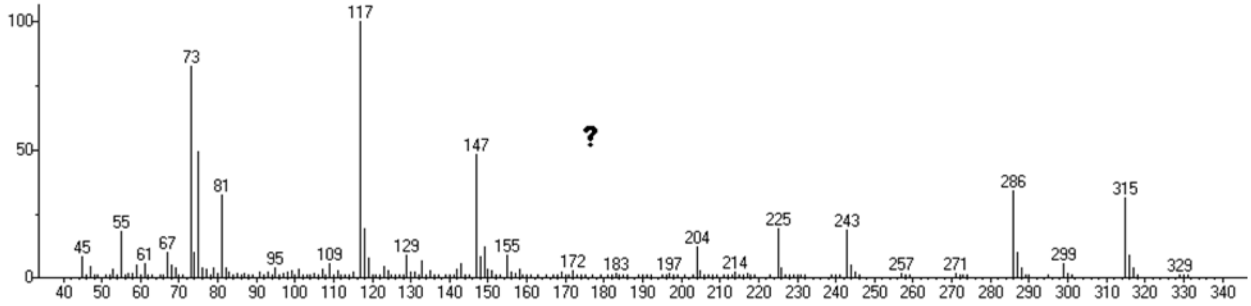

Compound 14 (18.08min): 10HDAA

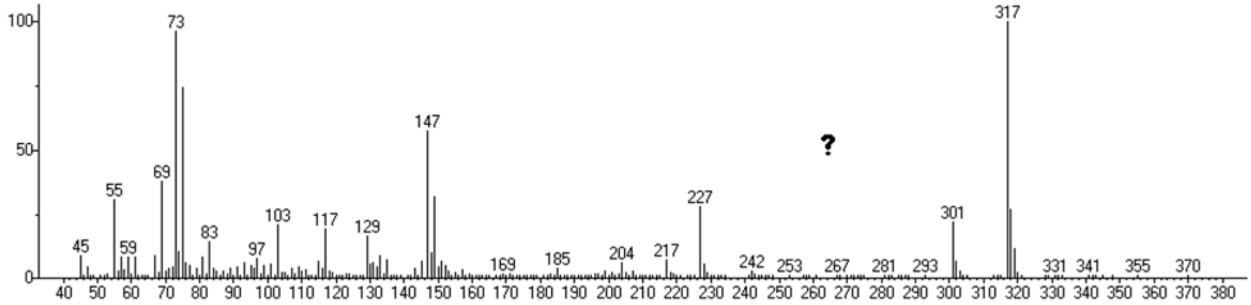

Compound 15 (18.29min): Unk7

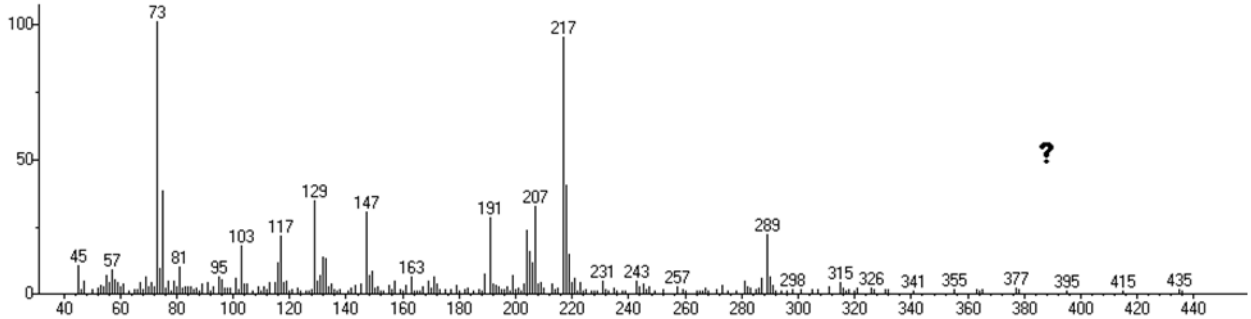

Compound 16 (19.12min): 10HDA

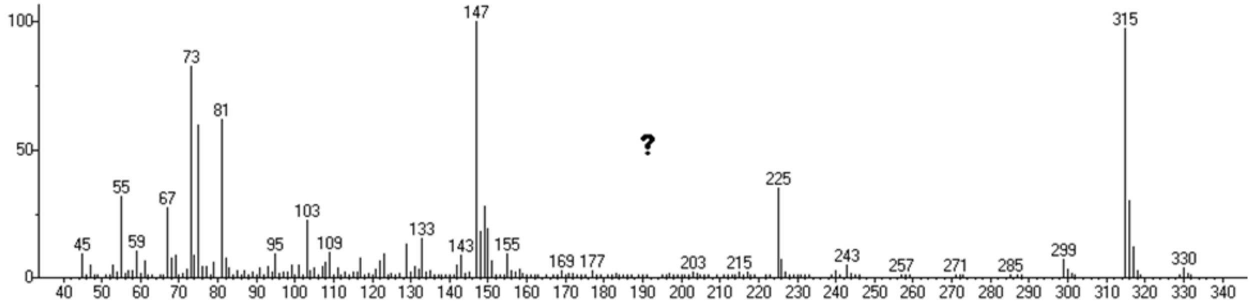

Compound 17 (19.75min): Decanedioic acid

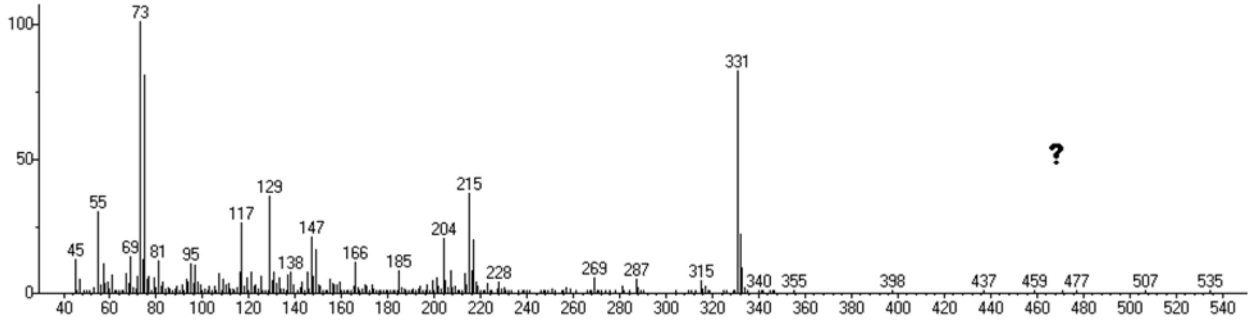

Compound 18 (19.87min):Unk8

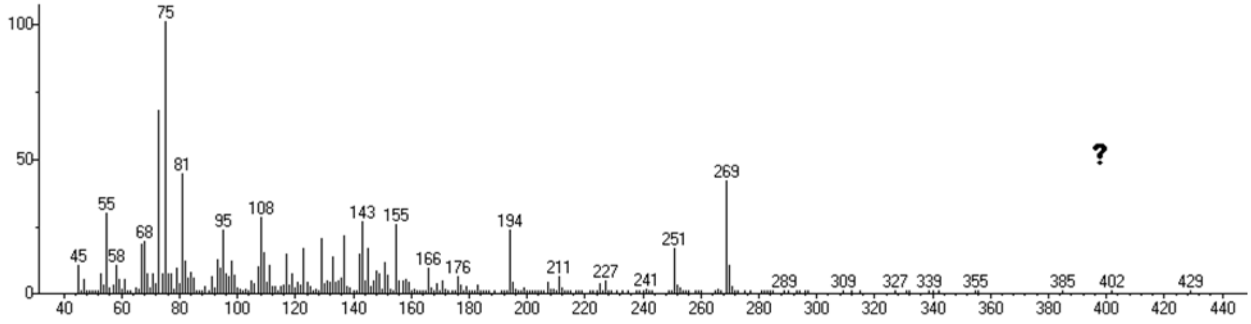

Compound 19 (20.58min): Unk9

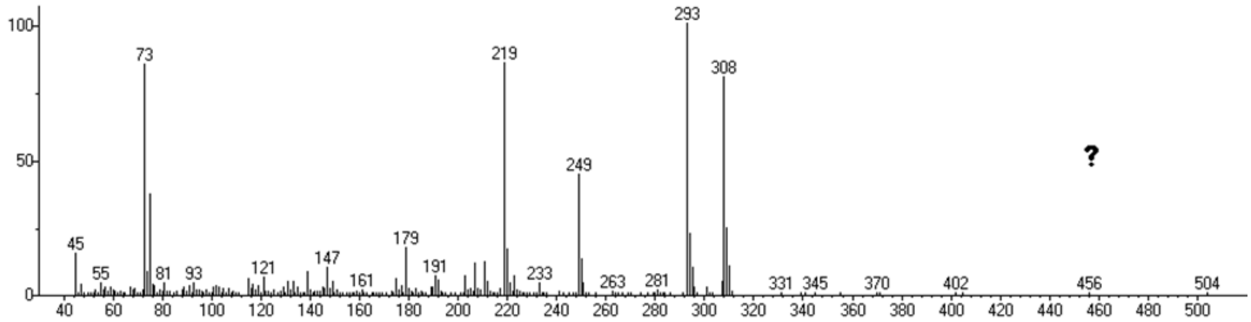

Compound 20 (20.76min): Unk10

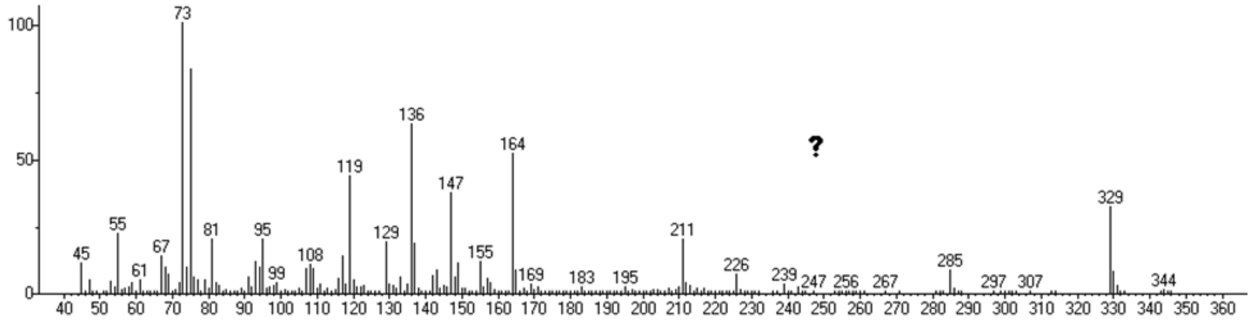

Compound 21 (20.86min): Unk11

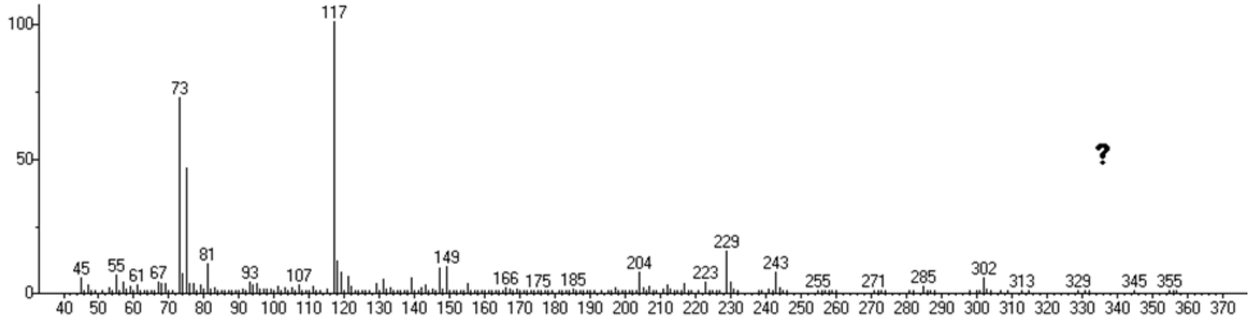

Compound 22 (21.53min): Unk12

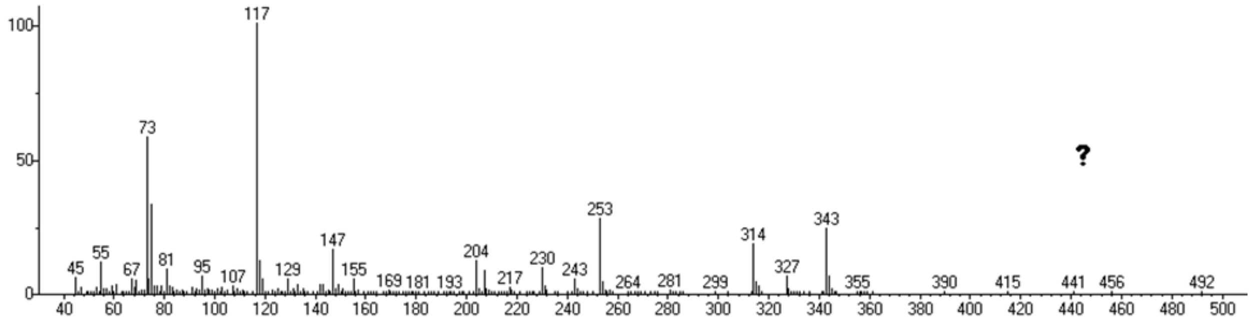

Compound 23 (21.78min): Unk13

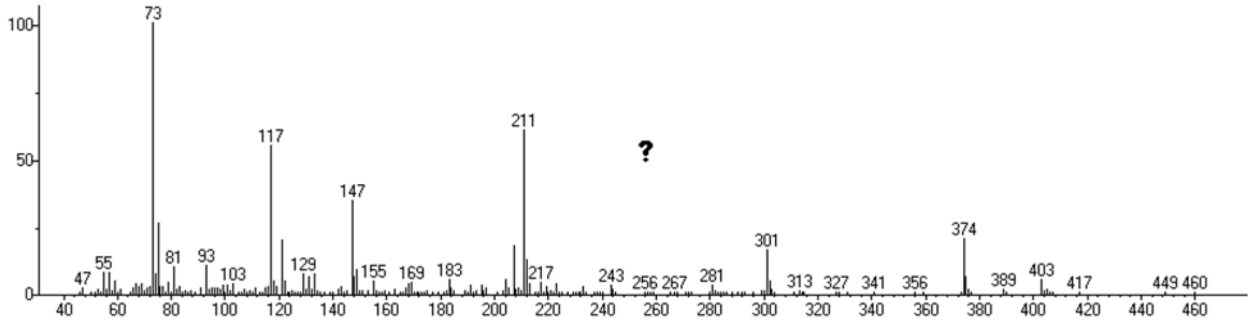

Compound 24 (22.11min): Unk14

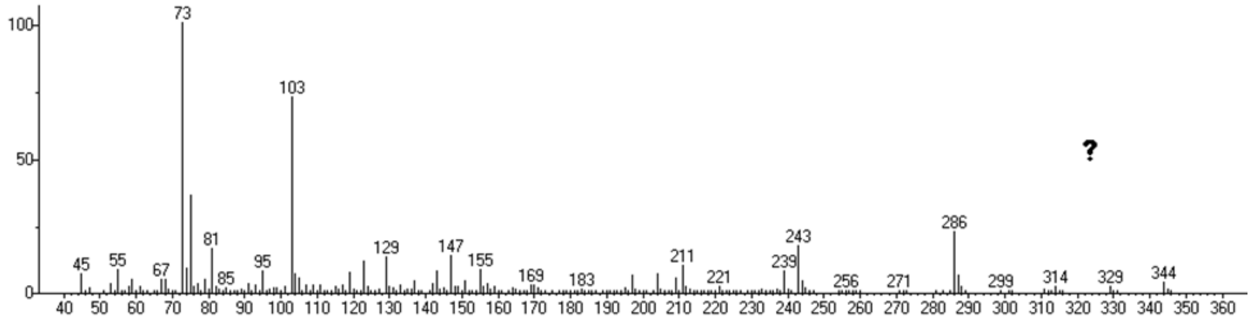

Compound 25 (23.15min): Unk15

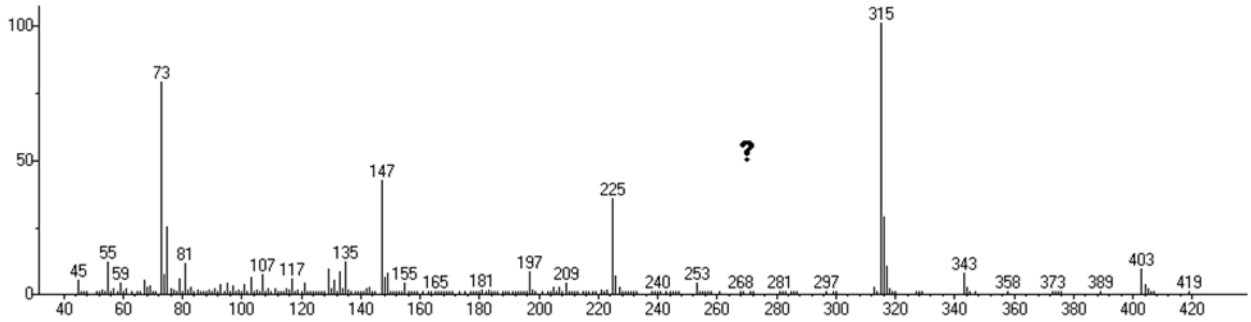

Compound 26 (27.47min): cis-13-octadecanoic acid

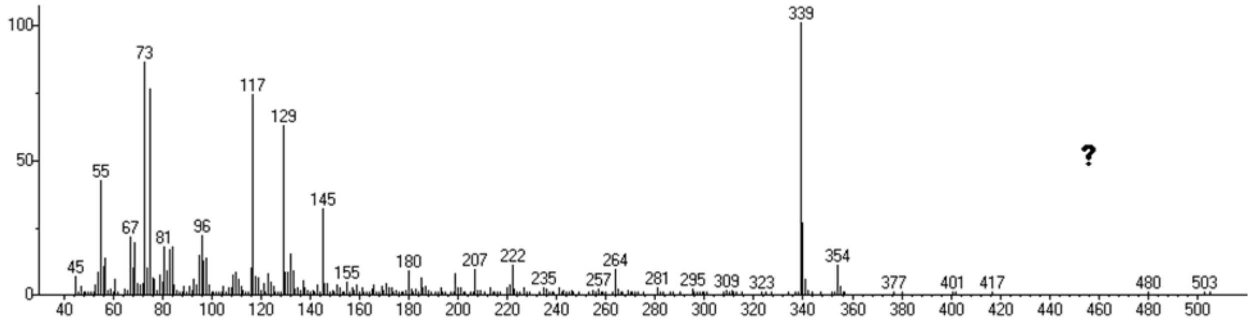

Compound 27 (28.08min): Octadecanoic acid

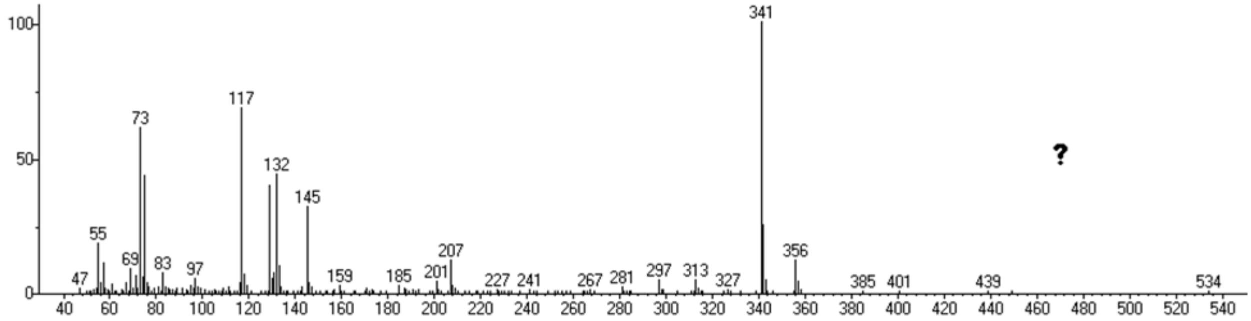

Supplement: Figure S1 — Fragmentation patterns of individual compounds in experiment 1. Shown here are the compounds recorded from the mandibular gland extracts of the queens exposed to CO2 and CO2+ physical manipulation (fragmentation patterns are shown for one of the CO2 queens). (PDF) [file pone.0078637.s001.pdf]
