# Supplementary material for: Chemical Profiles of Two Pheromone Glands Are Differentially Regulated by Distinct Mating Factors in Honey Bee Queens (Apis mellifera L.)
Source: PLoS One. 2013 Nov 13;8(11):e78637. doi: 10.1371/journal.pone.0078637 (PMC3827242; doi:10.1371/journal.pone.0078637)

Figure S2. Fragmentation patterns of individual compounds in experiment 2. Shown here are the compounds recorded from the mandibular gland extracts of the queens instrumentally inseminated with low or high volume of either saline or semen (fragmentation patterns are shown for one of the SA1 queens).

Compound 1 (12.34min): Unk1

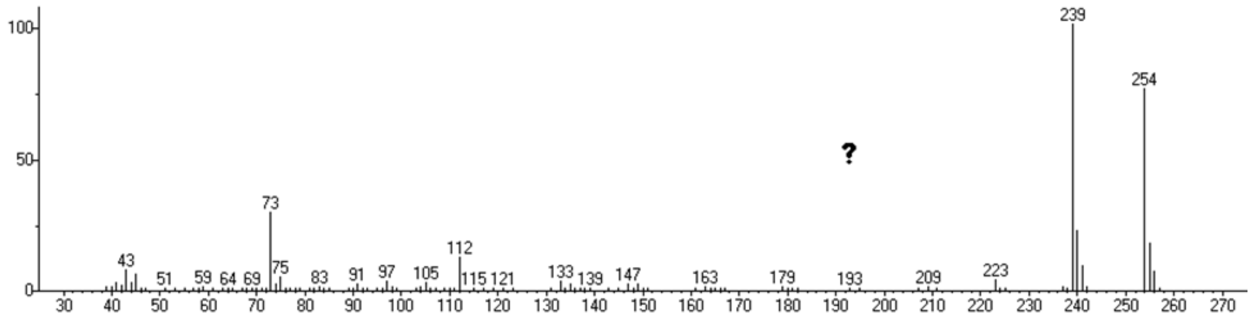

Compound 2 (13.34min): Unk2

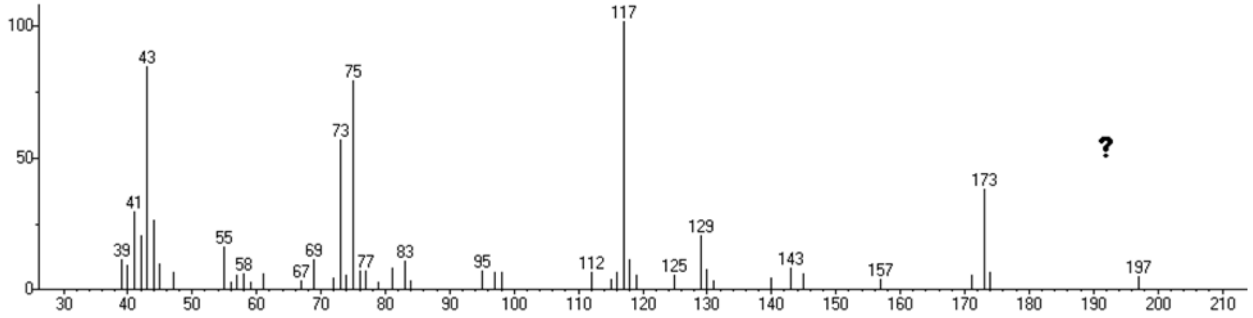

Compound 3 (14.45min): HOB

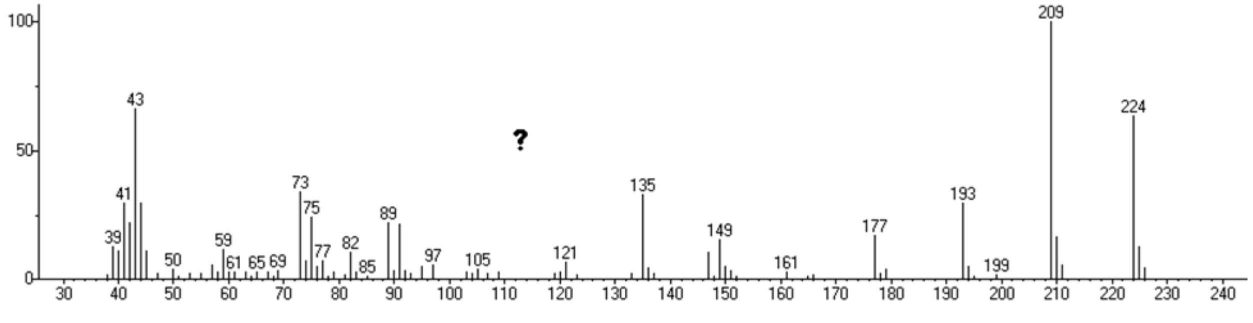

Compound 4 (16.23min): Unk3

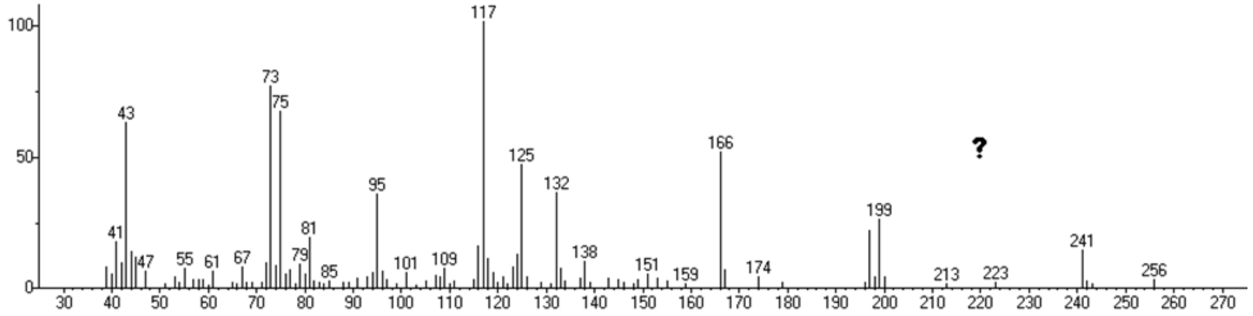

Compound 5 (17.24min): 7OH octanoic acid

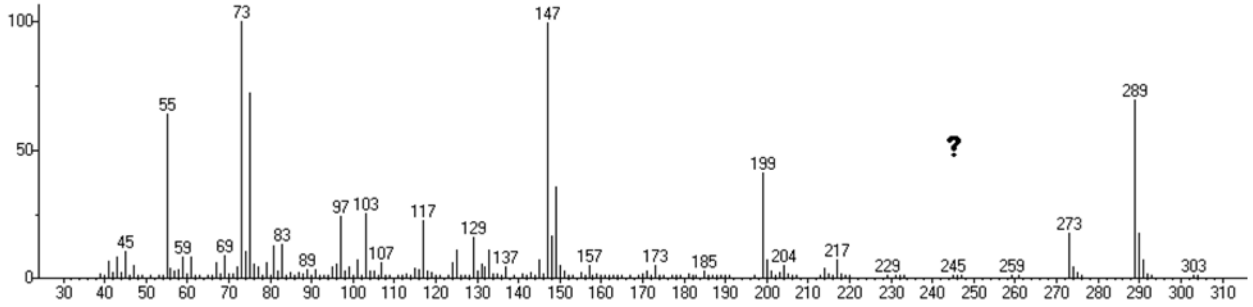

Compound 6 (17.47min): Benzoic acid

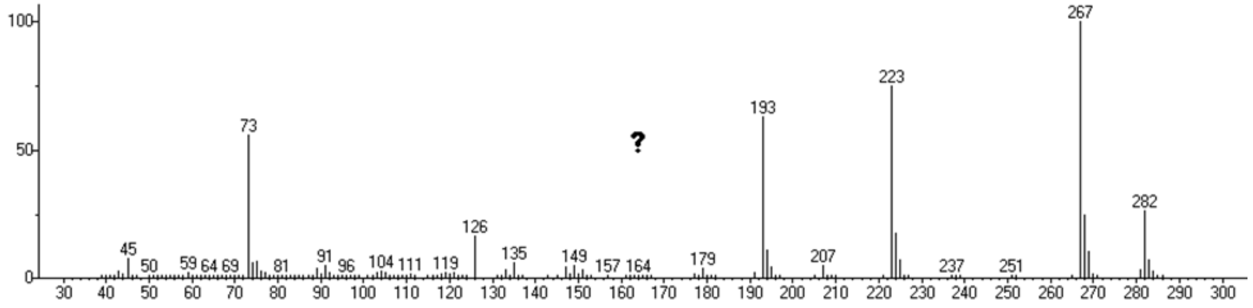

Compound 7 (18.05min): Unk4

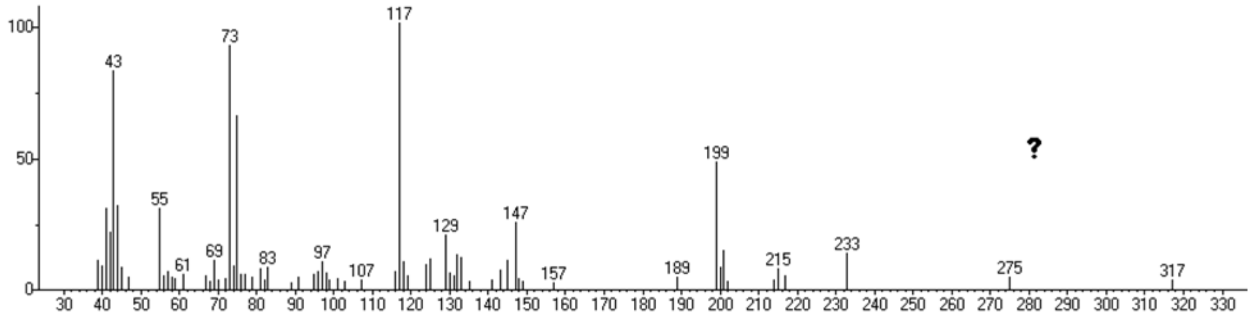

Compound 8 (18.28min): Unk5

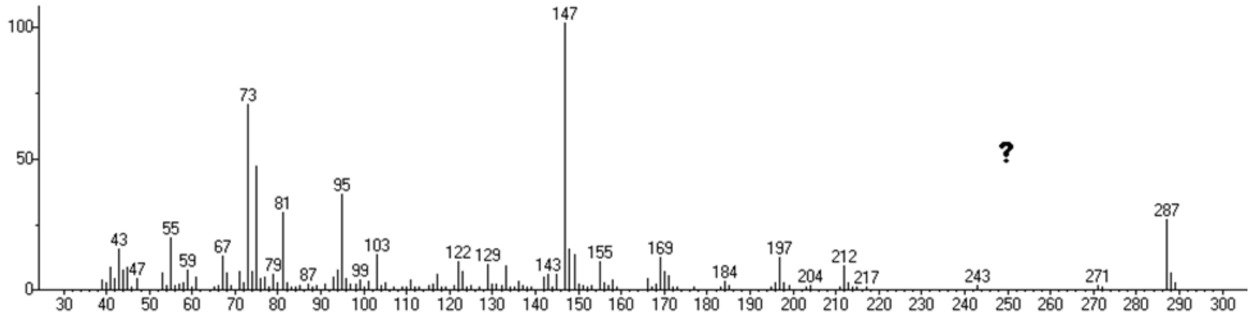

Compound 9 (19.18min): 9-ODA

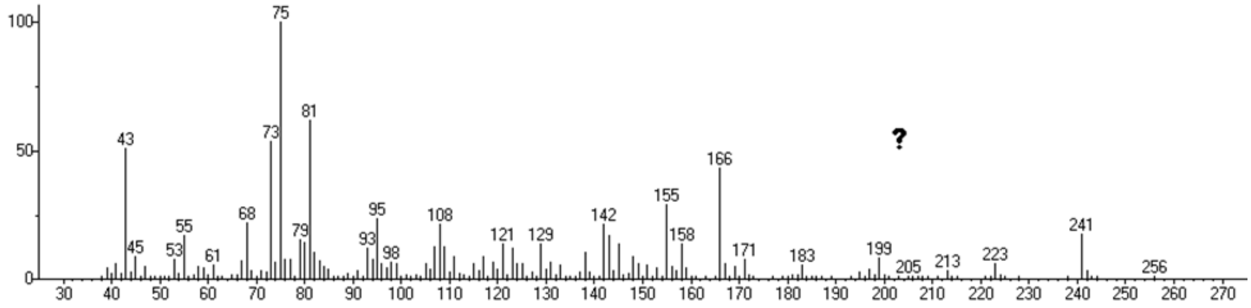

Compound 10 (19.32min): HVA

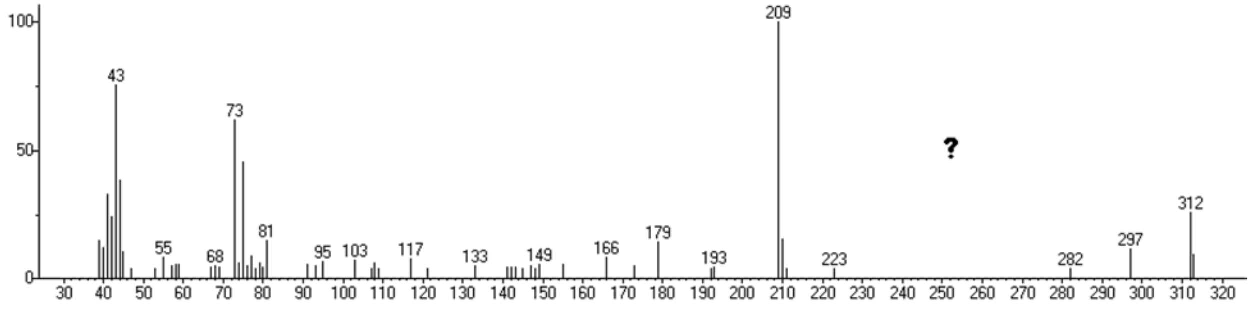

Compound 11 (19.83min): 9-oxydecanoic acid

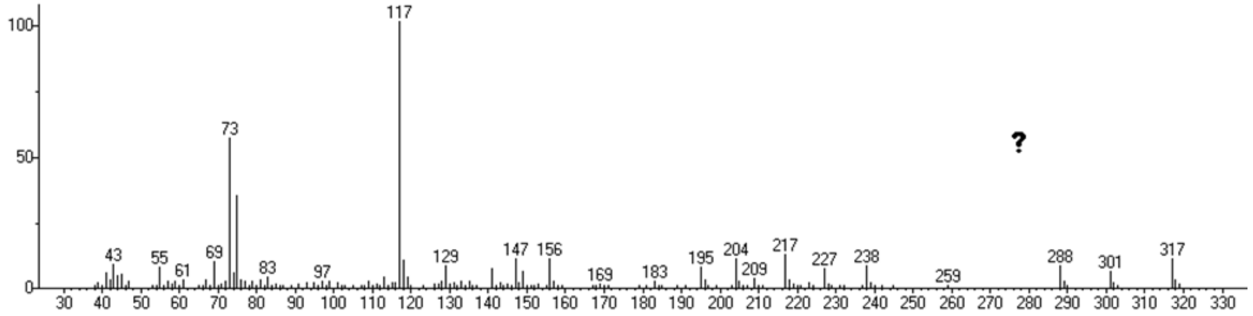

Compound 12 (20.56min): Unk6

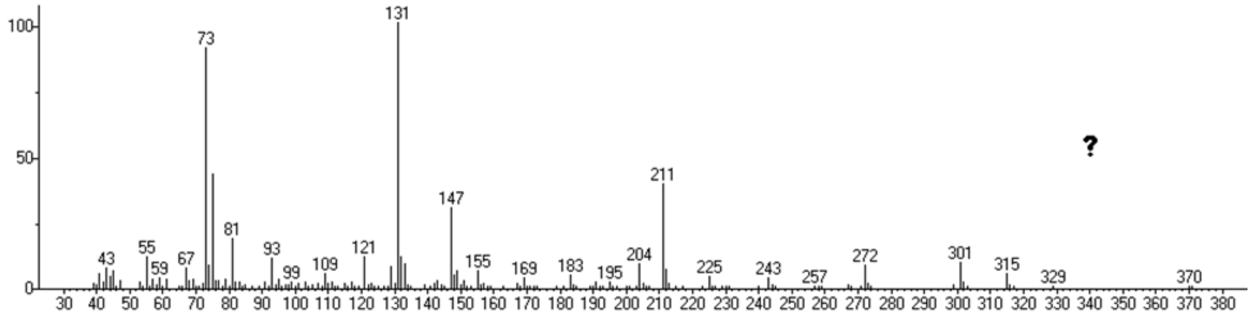

Compound 13 (20.92min): 9-HDA

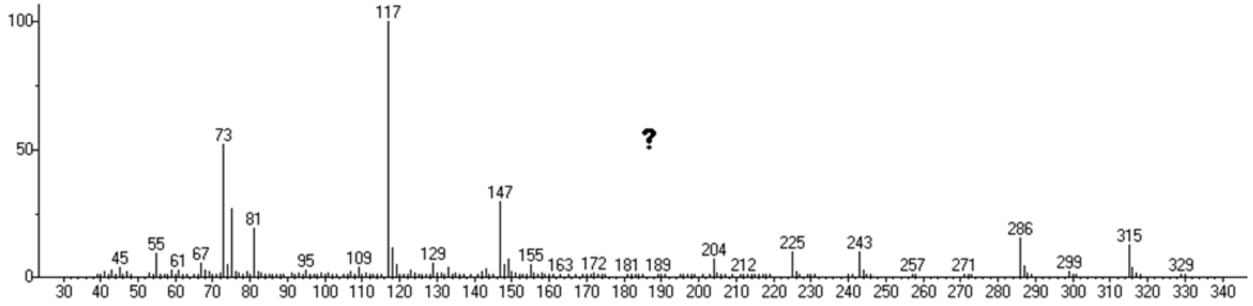

Compound 14 (21.03min): Unk7

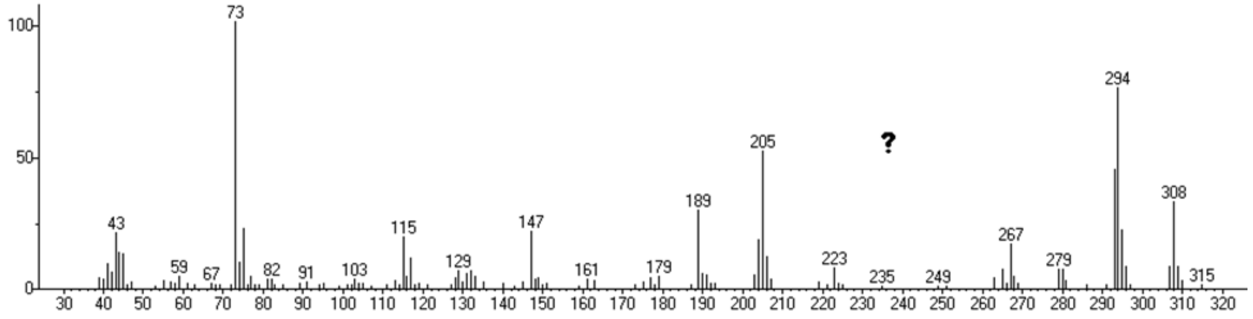

Compound 15 (21.28min): 10HDAA

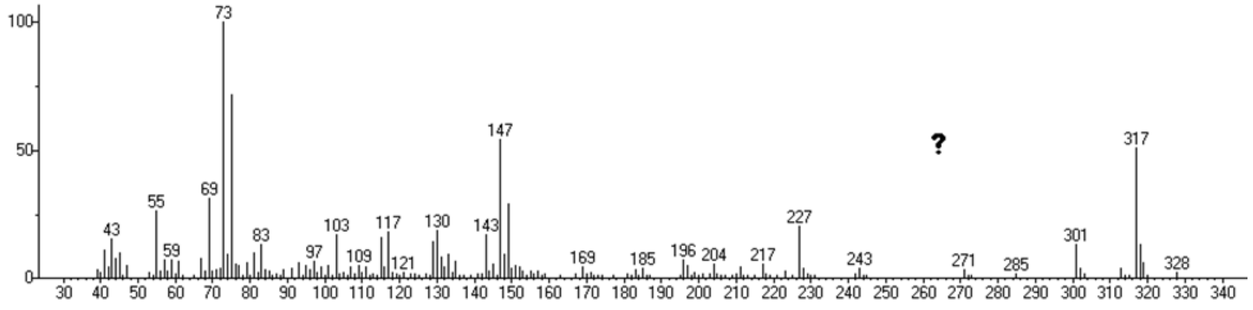

Compound 16 (22.08min): Tetradecanoic acid

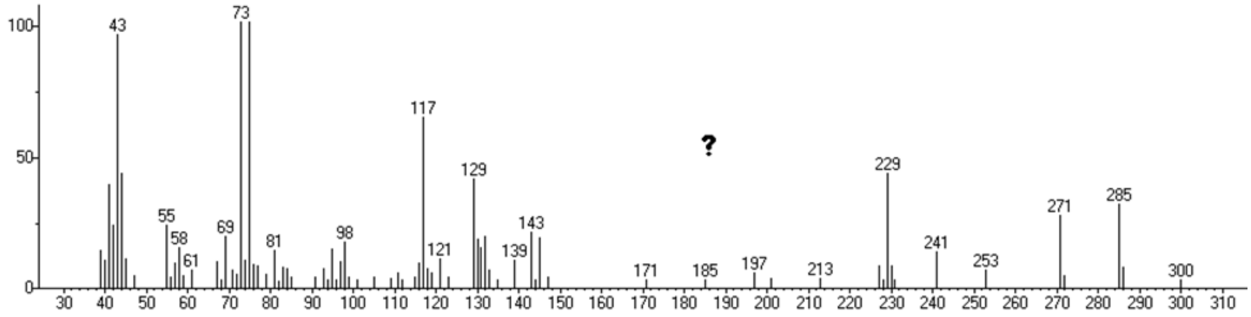

Compound 17 (22.43min): 10HDA

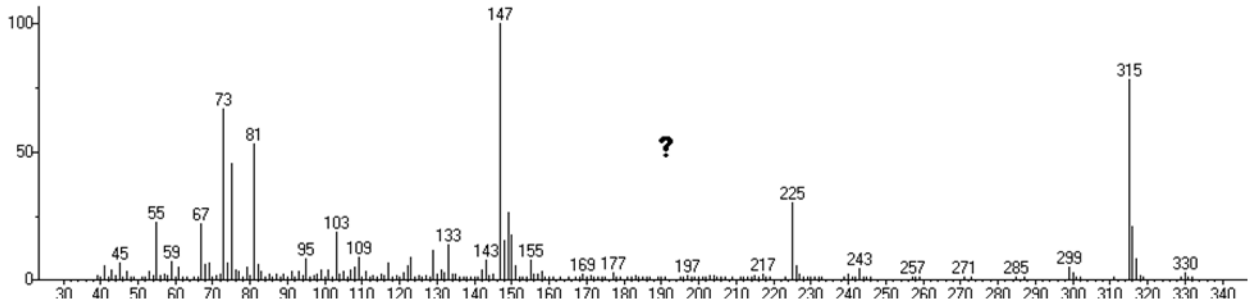

Compound 18 (22.74min): Unk8

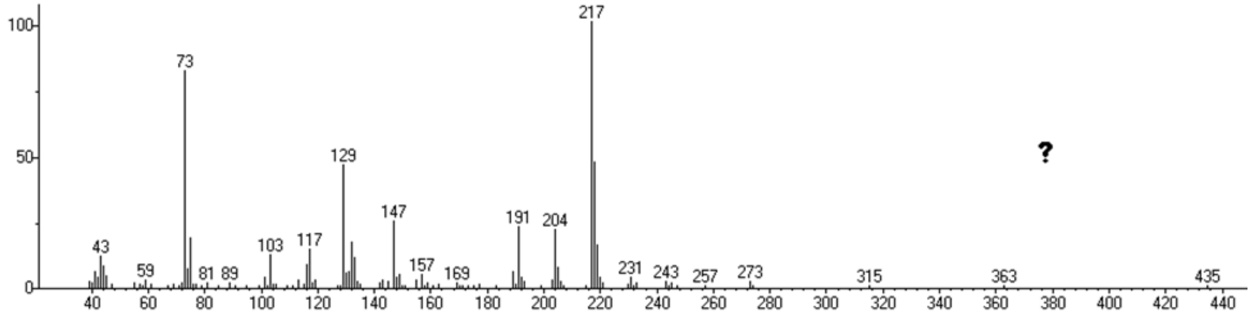

Compound 19 (23.22min): Decanedioic acid

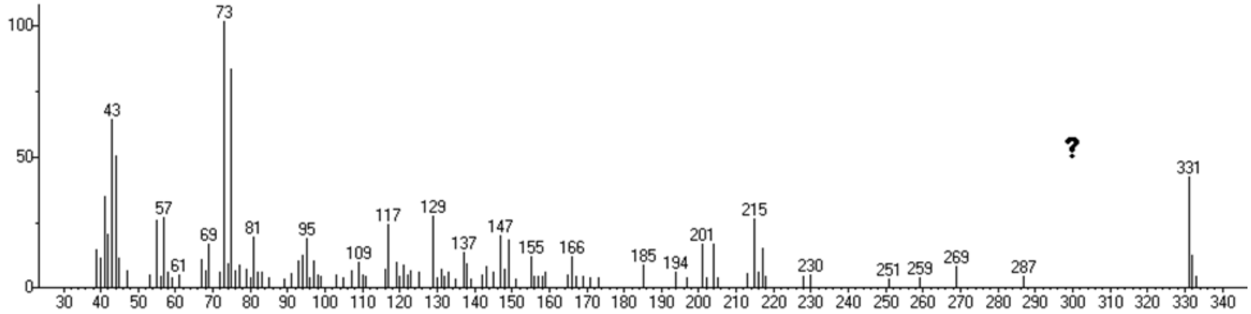

Compound 20 (23.44min): Unk9

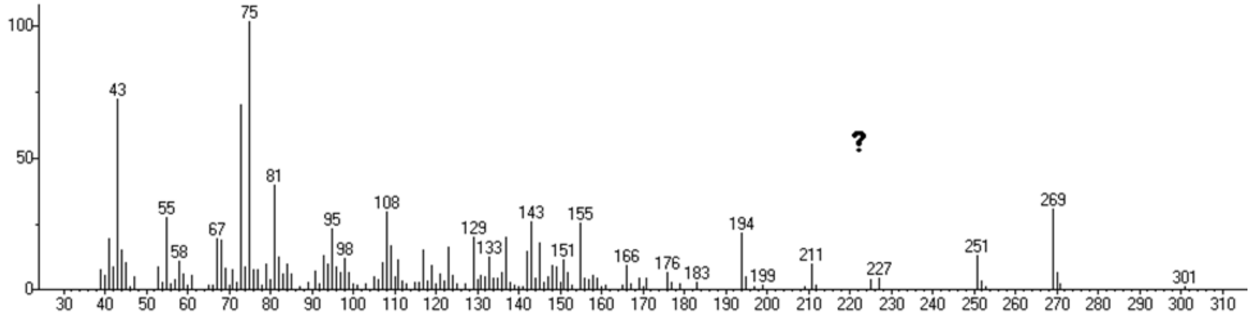

Compound 21 (24.65min): Unk10

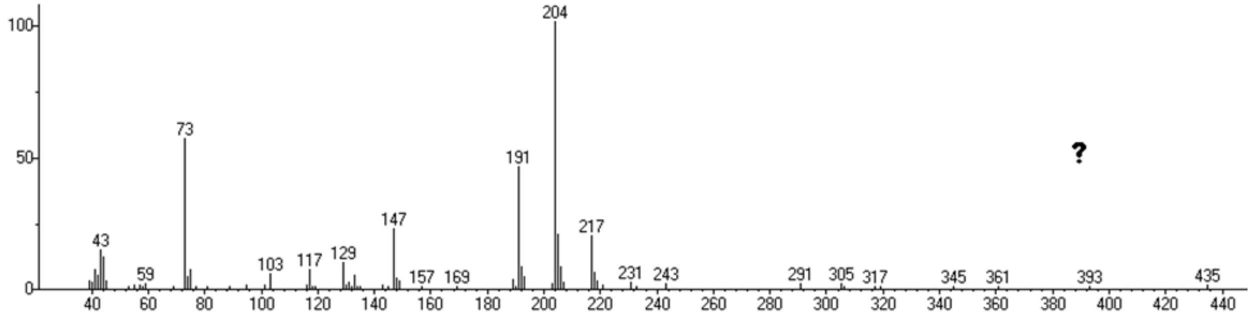

Compound 22 (25.55min): Unk11

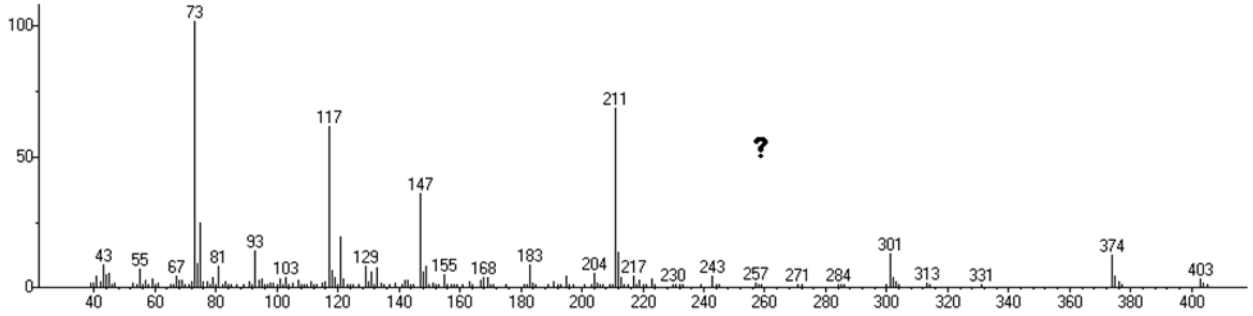

Compound 23 (25.86min): Unk12

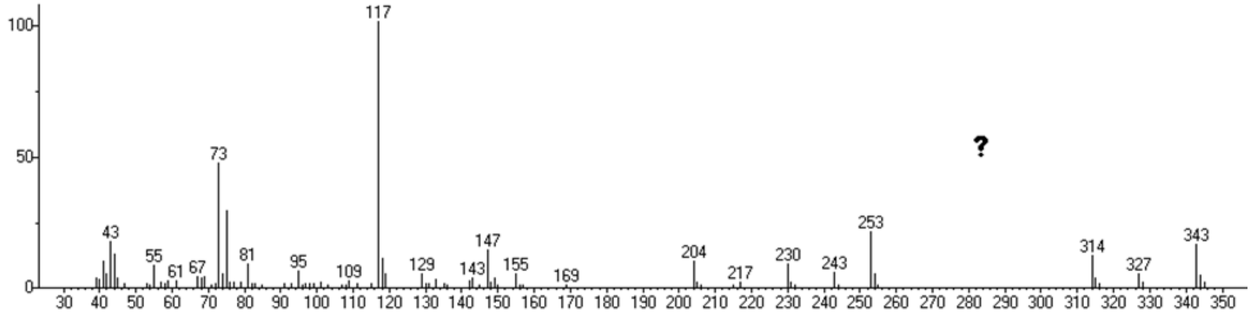

Compound 24 (26.23min): Unk13

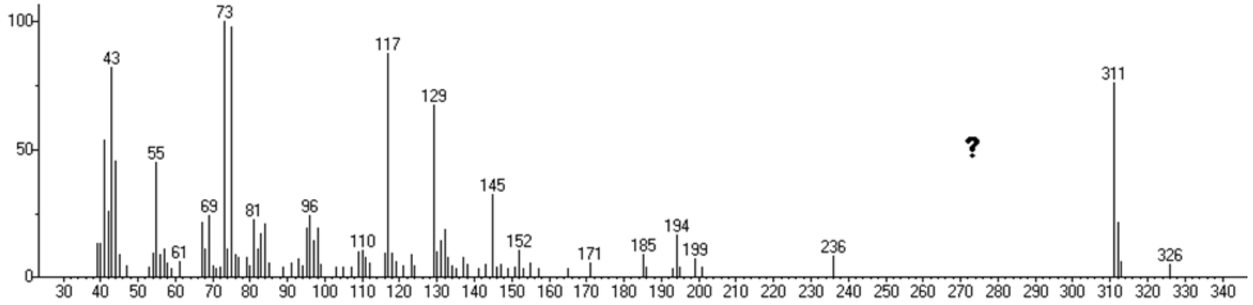

Compound 25 (26.75min): Unk14

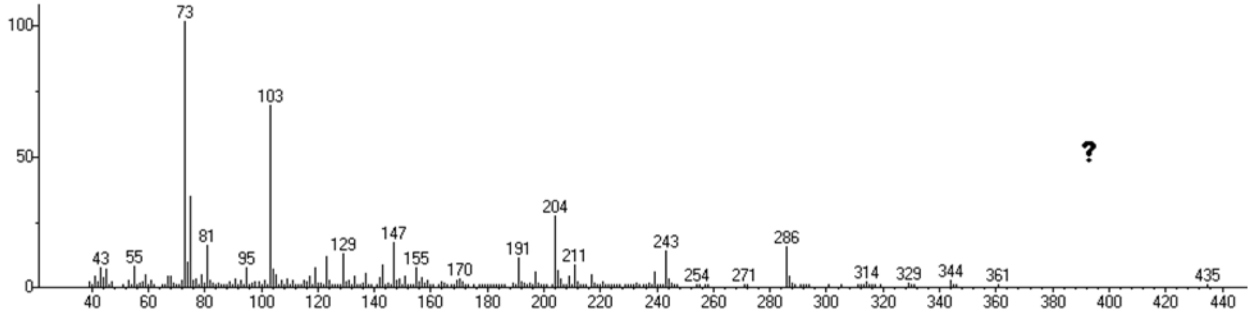

Compound 26 (27.80min): Unk15

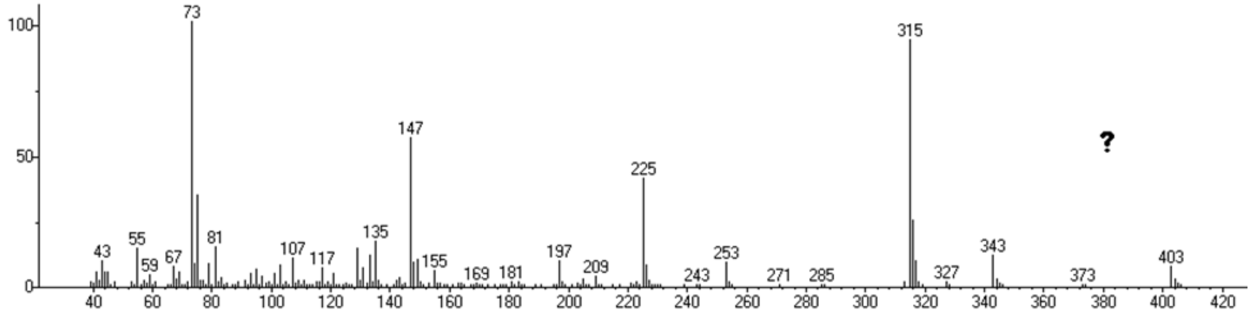

Compound 27 (30.76min): Octadecanoic acid

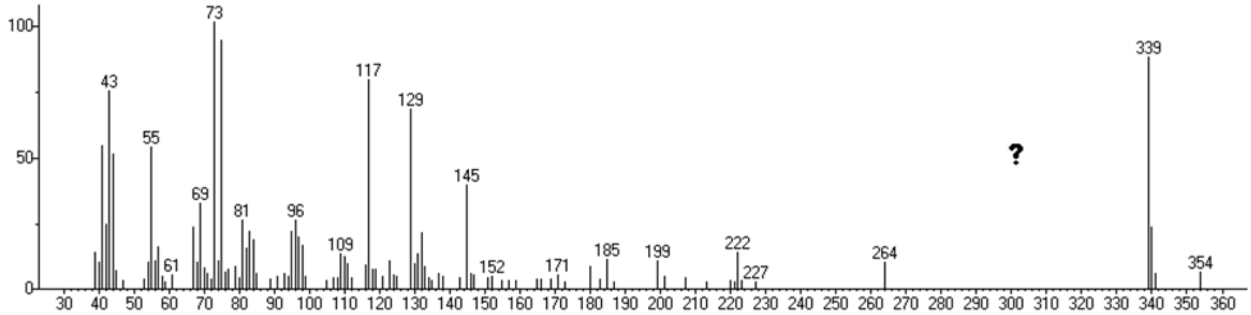

Compound 28 (31.14min): Unk16

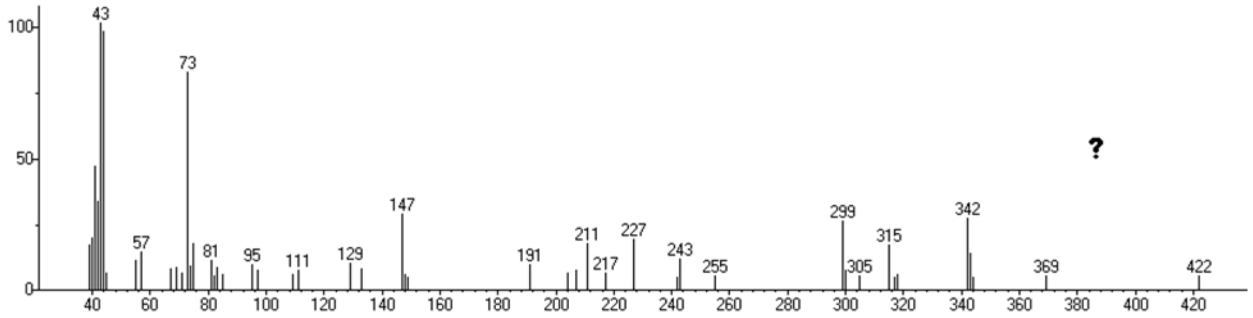

Supplement: Figure S2 — Fragmentation patterns of individual compounds in experiment 2. Shown here are the compounds recorded from the mandibular gland extracts of the queens instrumentally inseminated with low or high volume of either saline or semen (fragmentation patterns are shown for one of the SA1 queens). (PDF) [file pone.0078637.s002.pdf]
